# The integrated stress response protects against ER stress but is not required for altered translation and lifespan from dietary restriction in *Caenorhabditis elegans*

**DOI:** 10.3389/fcell.2023.1263344

**Published:** 2023-12-14

**Authors:** Zhengxin Ma, Jordan Horrocks, Dilawar A. Mir, Matthew Cox, Marissa Ruzga, Jarod Rollins, Aric N. Rogers

**Affiliations:** MDI Biological Laboratory, Bar Harbor, ME, United States

**Keywords:** integrated stress response, nonsense mediated decay, aging, dietary restriction, endoplasmic reticulum stress

## Abstract

The highly conserved integrated stress response (ISR) reduces and redirects mRNA translation in response to certain forms of stress and nutrient limitation. It is activated when kinases phosphorylate a key residue in the alpha subunit of eukaryotic translation initiation factor 2 (eIF2). General Control Nonderepressible-2 (GCN2) is activated to phosphorylate eIF2α by the presence of uncharged tRNA associated with nutrient scarcity, while protein kinase R-like ER kinase-1 (PERK) is activated during the ER unfolded protein response (UPR^ER^). Here, we investigated the role of the ISR during nutrient limitation and ER stress with respect to changes in protein synthesis, translationally driven mRNA turnover, and survival in *Caenorhabditis elegans*. We found that, while GCN2 phosphorylates eIF2α when nutrients are restricted, the ability to phosphorylate eIF2α is not required for changes in translation, nonsense-mediated decay, or lifespan associated with dietary restriction (DR). Interestingly, loss of both GCN2 and PERK abolishes increased lifespan associated with dietary restriction, indicating the possibility of other substrates for these kinases. The ISR was not dispensable under ER stress conditions, as demonstrated by the requirement for PERK and eIF2α phosphorylation for decreased translation and wild type-like survival. Taken together, results indicate that the ISR is critical for ER stress and that other translation regulatory mechanisms are sufficient for increased lifespan under dietary restriction.

## Introduction

Aging is associated with a decline in mechanisms governing cellular resilience. This decline results in increased susceptibility to the negative effects of nutrient imbalances and stress, disrupting essential processes such as proteostasis and energy metabolism, and contributing to the accumulation of misfolded proteins and cellular dysfunction (Butcher and Lord, 2004; Haigis and Yankner, 2010; Kyriakakis et al., 2015). In particular, impaired proteostasis is closely linked to the development of neurodegenerative disorders, such as Alzheimer’s, Parkinson’s, and Huntington’s diseases (Kim et al., 2022). Interventions that help maintain cellular homeostasis hold the potential to help treat such disorders.

The ISR is a conserved signaling pathway for translationally-mediated adaptation to various forms of stress that perturb cellular homeostasis (Pakos-Zebrucka et al., 2016). In recent years, the ISR has gained notoriety due to its role in neurodegenerative disease and possible role in aging (Bond et al., 2020; Derisbourg et al., 2021a; Derisbourg et al., 2021b). The key event in the ISR is phosphorylation of the eukaryotic translation initiation factor subunit (eIF) 2α, which inhibits the exchange of eIF2 GDP-to-GTP by eIF2B and thereby suppresses the translation-initiating ternary complex (TC). This complex is a crucial molecular component involved in the initiation of protein synthesis comprising eIF2, GTP and Methionyl-tRNA (Jackson et al., 2010). Inhibiting GDP-to-GTP recharging leads to global attenuation of most translation except for certain genes, notably those containing upstream open reading frames (uORFs) in the 5’ untranslated region, such as the activating transcription factor 4 (ATF4) (Costa-Mattioli and Walter, 2020). Under normal conditions, such transcripts are inefficiently translated and destabilized due to the presence of premature termination codons (PTC) associated with the uORFs.

In mammals, eIF2α is phosphorylated at S51 by one of four kinases depending on the stress encountered. These include GCN2 (general amino acid control nonderepressible 2), PKR (double stranded RNA-dependent protein kinase), HRI (heme-regulated inhibitor), and PERK (PKR-like ER kinase). When tRNAs are uncharged, as happens during amino acid deprivation, GCN2 activates the ISR, reducing and redirecting protein synthesis to conserve energy and resources (Lee et al., 2008; Bunpo et al., 2010; Jonsson et al., 2019). PERK activates the ISR during ER stress when proteins are misfolded or when there are perturbations in calcium as a part of the unfolded protein response (UPR)^ER^ (Korennykh and Walter, 2012; Wang and Kaufman, 2016). PKR is involved in antiviral defense activated by double-stranded RNA (Harding et al., 2000; Barber, 2001), whereas HRI is a heme sensor that reduces globin synthesis during heme deficiency (Chen, 2014). In each case, phosphorylation of eIF2α results in rapid attenuation of mRNA translation and redeployment of remaining translation to stress responsive genes (Harding et al., 2000; Teske et al., 2011).

The *Caenorhabditis elegans* genome only encodes two ISR activators. These are GCN-2 (GCN2) and PEK-1 (PERK), which target eIF-2α at S49. Research on the ISR in *C. elegans* has provided valuable insights into the regulatory mechanisms and functional significance of the ISR. The amino acid sequence of eIF-2α shares nearly 50% identity with the human ortholog and the key phosphorylation site is conserved (Derisbourg et al., 2021a). ER stress triggered by overexpression of the glutamine-fructose 6-phosphate aminotransferase homolog *gfat-1* increases the ISR-dependent expression of ATF-4 (Horn et al., 2020), indicating that the key ISR components are conserved. The kinase PEK-1 is essential to protect larvae against bacterial infection (Richardson et al., 2011) and transition into dauer state under ER stress (Kulalert and Kim, 2013). In addition, GCN-2 mediates mitochondrial stress in *C. elegans*, whereas HRI is responsible for ISR activation under mitochondrial stress in mammals (Baker et al., 2012).

The ISR may play a role in the effects of dietary restriction (DR), which increases healthy lifespan by reducing and redirecting mRNA translation. Many types of DR exist, including caloric restriction, restriction of specific macronutrients or micronutrients, and intermittent fasting (Greer and Brunet, 2009). In *C. elegans*, dilution of their bacterial food source is a frequently used method of DR, increasing lifespan by 40% (Rollins et al., 2019). Research on understanding how changes in mRNA translation mediate beneficial effects on health and longevity has focused mostly on anabolic machinery controlled by the nutrient-sensing mechanistic Target of Rapamycin (mTOR). Little is known about how mTOR-independent ISR contributes to mRNA translation changes important for increasing healthy lifespan under DR.

Healthy lifespan is also governed by the proteostasis-regulating UPR^ER^, which governs translation changes through PERK/PEK-1 and the ISR. A variety of internal and external stimuli can cause ER stress that overlap with the ISR, including hypoxia, nutrient scarcity, and pathogen infection (Wang and Kaufman, 2016). When misfolded proteins accumulate in the ER lumen, it creates proteotoxic stress that triggers the UPR^ER^, which is controlled by three transmembrane proteins comprising transcription factor 6 (ATF6), inositol-requiring enzyme 1 (IRE1), and PERK. Once activated, these sensors initiate distinct signaling pathways to regulate gene expression both transcriptionally and translationally and restore protein-folding capacity (Ron and Walter, 2007; Wek and Cavener, 2007). Loss of PERK is associated with disturbed ER morphology and calcium signaling (Verfaillie et al., 2012). Pathologies like cell death, progressive diabetes mellitus, and exocrine pancreatic insufficiency are observed in *PERK*-deficient mice (Harding et al., 2001). Loss of the *C. elegans* ortholog, *pek-1*, leaves animals relatively healthy under standard conditions and partial knockdown of *pek-1* with RNAi does not reduce survival of wild-type *C. elegans* during ER stress (Howard et al., 2016). However, a study using a *pek-1* mutant shows that this gene may be important for survival under ER stress in *C. elegans* (Derisbourg et al., 2021b).

Translation initiation regulates longevity and is controlled by two complexes. One comprises the cap-binding complex downstream of mTOR, which circularizes mRNA and helps recruit additional translation factors (Kapahi et al., 2010). mTOR is a nutrient sensor that can act independently or in parallel to the ISR and other initiation-regulating complex, the TC (Sonenberg and Hinnebusch, 2009). Studies showed that amino acid depletion extends the lifespan in yeast and activates the ISR, but there is no direct evidence proving that the ISR is essential for the longevity benefit associated with this form of DR (Jiang et al., 2000; Ecker et al., 2010; Kaya et al., 2015). Interestingly, it was found that abrogating the ISR in phosphorylation-defective eIF-2α mutants increases the lifespan of *C. elegans*, suggesting that chronic inhibition of the ISR favors protein homeostasis in the absence of stress (Derisbourg et al., 2021b).

The ISR and nonsense-mediated RNA decay (NMD) pathways are connected via translation. Concurrent with, and dependent on ribosome translocation during protein synthesis, NMD degrades aberrant mRNA containing PTCs. The aberrant mRNA includes mRNA bearing retained introns or those containing uORFs in the 5’ UTR of genes translationally activated by the ISR. Thus, it serves as an mRNA quality control mechanism but also regulates a significant proportion of regular (i.e., non-PTC bearing) mRNAs through mechanisms that are not well characterized (Hug et al., 2016). Under hypoxic stress, NMD is inhibited in an ISR dependent manner in mammalian tissue culture. Interestingly, this was found to favor stabilization of certain stress-induced “normal” (i.e., not bearing PTCs) mRNAs normally targeted by NMD under non-stressed conditions (Gardner, 2008). Besides hypoxia, ER stress and amino acid starvation also trigger the escape of stress-associated mRNA that are usually targeted by NMD, including the ISR effector ATF4 (Karam et al., 2015; Martin and Gardner, 2015; Li et al., 2017). Under nutrient deprivation stress associated with DR, overall NMD activity is diminished, but factors mediating its regulation are required for lifespan extension (Rollins et al., 2019)**.** Whether the ISR is essential to alter NMD and promote longevity associated with DR remains unclear.

Here, we investigated the role of the ISR in translation and lifespan extension under DR in *C. elegans.* We validated that DR activates the ISR by GCN-2 but found that eIF-2α phosphorylation is not required for lifespan extension and downregulation of protein synthesis. Additionally, changes in the prevalence of NMD targets are not ISR dependent under nutrient-limited conditions. Furthermore, while the ISR is important for ER stress adaptation, it is not necessary to respond to starvation and heat stress. Interestingly, while neither *pek-1* or *gcn-2* genes are individually required for increased lifespan under DR, double mutants show a complete loss of the lifespan phenotype under this condition.

## Materials and methods

### *Caenorhabditis elegans* strains and culture

All strains used are listed in Supplementary Table S1. The worms were cultured at 20°C and maintained on nematode growth medium (NGM) plates seeded with *Escherichia coli* OP50 unless indicated otherwise. All worms grew at least three generations under normal, unstressed conditions prior to use in experiments. Mutant strains were obtained from the CGC and backcrossed with the wild type Bristol N2 strain at least three times.

### Lifespan analysis

*Caenorhabditis elegans* were developmentally synchronized from a 4 h egg lay and transferred to 10 cm peptone-free nematode growth media (NMG) plates spotted with 10^11^ CFU/mL (AL) or 10^9^ CFU/mL (DR) OP50 upon reaching day 1 of adulthood. Plates had 25 mg/mL of carbenicillin to prevent bacterial growth. The worms were transferred daily to fresh plates until they reached the post-reproductive stage and then transferred every 4 days. The animals were scored for survival every other day by monitoring the movement after tapping the worm gently with a platinum wire.

### Heat stress recovery

Synchronized worms were transferred to fresh plates daily for 1 week and then shifted to 35°C for 4 h. After the heat shock, the nematodes were monitored daily for survival.

### ER stress induction

NGM plates were spotted with OP50. After 1 day, either DMSO or 25 μg/mL tunicamycin dissolved in DMSO was added to each plate. Another day was allowed to pass before animals were exposed to treated agar plates. Due to the large number of animals required for experiments involving polysome profiling, animals were synchronized by bleaching gravid adults. In translation and eIF-2α phosphorylation experiments, synchronized adults were transferred to tunicamycin or DMSO plates for 3 h and washed 3 times in M9 prior to analysis. To minimize vulval ruptures in survival assays, adult worms were treated with 50 μg/mL 5-fluoro-2-deoxyuridine (FUDR) for 2 days prior to exposure to tunicamycin or DMSO.

### Polysome profiling

150 µL of pelleted day 1 adult worms were lysed on ice in 350 µL of solubilization buffer (300 mM NaCl, 50 mM Tris–HCl [pH 8.0], 10mM MgCl_2_, 1 mM EGTA, 400 U RNasin/mL, 3.55 µM cycloheximide, 1% Triton X-100, 1 mM PMSF, 1 mini tablet of protease inhibitor [Thermo Sicentific] and 0.1% sodium deoxycholate). An additional 200 µL of solubilization buffer was added after homogenization and the samples were incubated on ice for 1 h. Lysates were centrifuged at 14,000 × *g* at 4°C for 5 min and the supernatants were collected. 300 μL of each sample was loaded to the top of a 5%–50% sucrose gradient in high salt resolving buffer (140 mM NaCl, 25 mM Tris–HCl [pH 8.0], and 10 mM MgCl_2_) and centrifuged in a Beckman SW41Ti rotor at 38,000 × *g* at 4°C for 2 h. Gradient fractionation was conducted with a Piston Gradient Station equipped with a Triax flow cell (BioComp Instruments) and the absorbance was monitored at 260 nm continuously. Polysome profile quantification was carried out using R with codes adapted from Shaffer and Rollins (2020).

### SUnSET puromycin assay

Bacteria were spotted into 12-well plates containing 2 mL of peptone-free NGM agar. Day 1 adults were assigned to AL (*ad libitum*, i.e., full fed), fasting (no bacteria, FT), or AL supplemented with 10 mM cycloheximide and incubated for 3 h. 300 μL of 1 mM puromycin was added to each well and incubated for 1 h. The worms were washed three times with M9 and 50 µL of worm slurry was frozen immediately in liquid nitrogen. To extract protein, worms were lysed on ice in 100 µL of solubilization buffer (300 mM NaCl, 50 mM Tris–HCl [pH 8.0], 10mM MgCl_2_, 1 mM EGTA, 1% Triton X-100, 1 mini tablet of protease inhibitor, and 1 mM PMSF) for 1 h and centrifuged at 14,000 × *g* at 4°C for 5 min. The Western blotting was conducted as described in the next section.

### Western blotting

Animals used in experiments were Day 1 adults. Worm collection, lysis, and protein extraction were performed as described in the “SUnSET puromycin assay” section. For SDS-PAGE, 20 µg of total protein as determined by Bradford protein assay was loaded on 4%–20% mini-Protean TGX stain-free gels (Bio-Rad) and separated at 100 V for 5 min followed by 150 V for 1 h. The SDS-PAGE gels were exposed to UV for 2.5 min for total protein quantity detection and then transferred to PVDF membranes. The membranes were incubated with primary antibody in EveryBlot blocking buffer (Bio-Rad) at 4°C overnight and further incubated with secondary antibody for 1 h after washing. The protein density was quantified using ImageJ 1.47V and normalized by the total quantity of protein. For puromycin assays, anti-puromycin Ab (clone 12D10, Sigma; 1:5000) was used followed by anti-mouse secondary Ab. For eIF2α S49 phosphorylation detection, the antibodies were anti-phospho-eIF2α rabbit polyclonal Ab (1:3000 dilution; Cell Signaling) and anti-rabbit secondary Ab. Three replicates were performed.

### NMD reporter and worm imaging

The NMD reporter strain PTCXi was described previously (Longman et al., 2007). Larval 4 stage worms were added to AL, DR or *smg-2* RNAi (Y48G8AL.6; Vidal library) (Rual et al., 2004) plates and transferred daily in the same conditions for 3 days. Fluorescence microscopy was performed with individual *C. elegans* using a Leica M165FC stereo microscope in the GFP channel (narrow band filter set, excitation ET470/40 nm, emission ET510/10 nm). Each worm was imaged on a 1% agarose pad on a glass slide and immobilized with a drop of 20 mM levamisole. Quantification of fluorescence was conducted using ImageJ for mean pixel intensity after correcting for background fluorescence. At least 15 worms were included in each replicate. Three replicates were performed.

### qPCR analysis

Adult worms were treated as described and then collected in TRIzol reagent (Invitrogen). RNA extraction and purification were performed using RNA Clean and Concentrator kit (Zymo Research) according to the manufacturer’s protocol followed by cDNA synthesis (QuantiTect Reverse Transcription kit [Qiagen]). qPCR analysis was conducted in technical triplicate using KAPA SYBR FAST qPCR Master Mix on a LightCycler 480 (Roche Applied Science, Indianapolis, IN, USA). Primers used are listed in Supplementary Table S2. The housekeeping gene *cdc-42* was used for target gene mRNA normalization. Gene expression changes were analyzed by the 2^−ΔΔC^_T_ method. Three replicates were conducted.

### Development and fecundity

Worms were synchronized by a 1-h timed egg lay. Time to reproductive development was measured from the time an egg was laid until it became an egg-laying adult. The fecundity of nematodes was determined by counting hatched progeny. Both development and fecundity assays were conducted at 20°C. At least 12 worms were included in each replicate. Three replicates were performed.

## Results

### Fasting upregulates eIF-2α phosphorylation that depends on GCN-2

**FIGURE 1:**
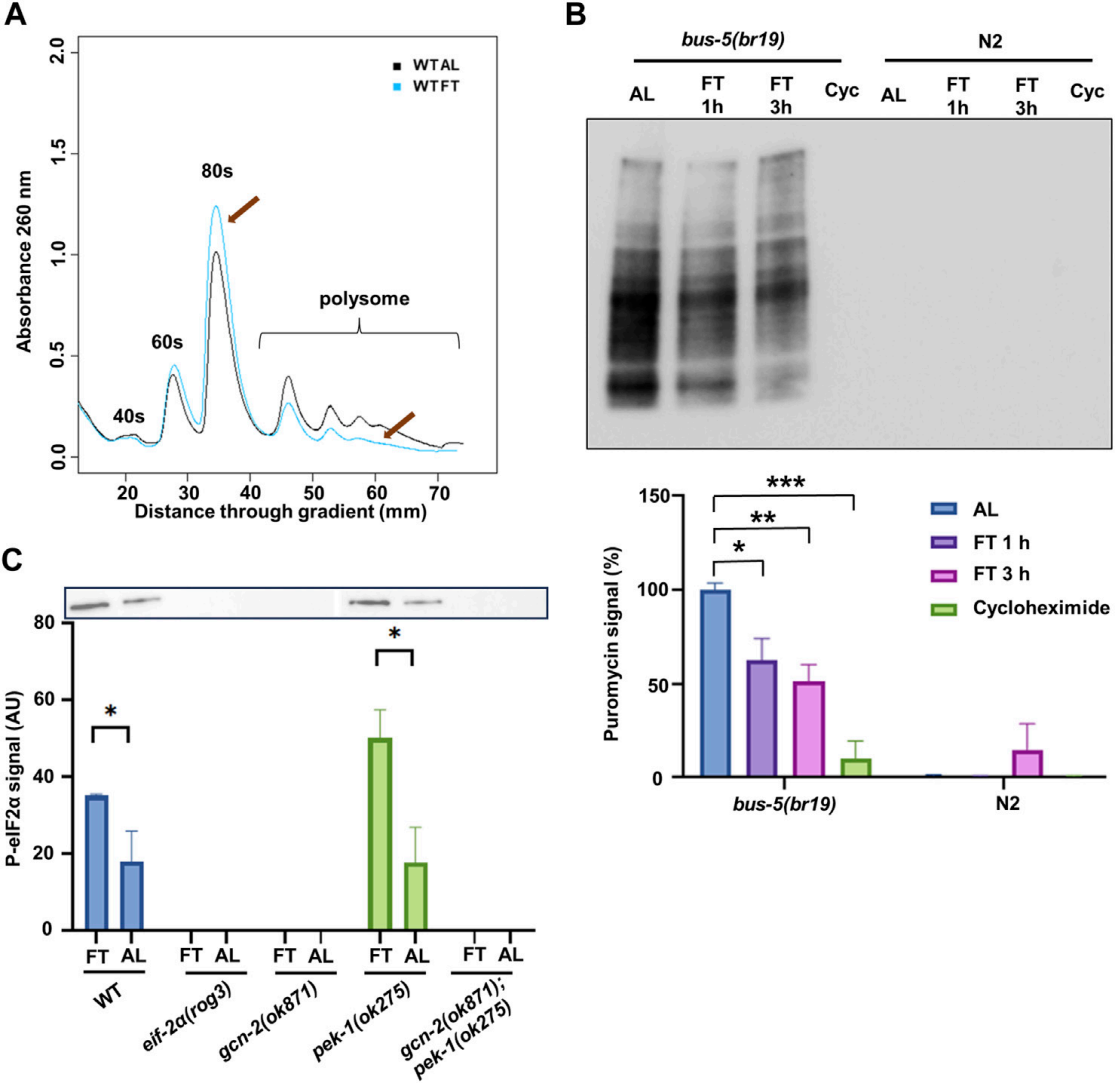
DR reduces translation and activates the ISR via GCN-2. **(A)** Polysome profiling of day 1 adult N2 with *ad libitum* feeding (AL) or fasting (FT) for 3 h. Representative of 3 replicates is shown. Arrows indicate the change of monosome and polysome. **(B)** SUnSET puromycin assay using Western blot with anti-puromycin antibodies to detect translation change in *bus-5(br19)* and N2 under AL, 1 h FT, 3 h FT and 10 mM cycloheximide treatments. Representative membrane is shown. The lower panel shows analysis from 3 experiments. **(C)** Western blot with anti-eIF2α phosphorylation antibody performed on day 1 adult under AL or FT for 3 h. Representative membrane is shown. Quantification was conducted with 3 independent experiments. Error bars represent means ± SEM, *t*-test was performed within each strain (*p < 0.05; **p < 0.01; ***p < 0.001).

In order to understand the role of ISR in response to nutrient deprivation, we started by assessing changes in translation. Polysome profiling provides an assessment of translation based on ribosome association with mRNA. A redistribution of ribosomes from high translation polysomes (i.e., mRNA bound by 2 or more ribosomes) to low translation monosomes was observed on young adult N2 worms after fasting (FT) for 3 h compared to full fed (*ad libitum*, AL) (Figure 1A). Quantitatively, the relative abundance of polysomes decreased by 20.3% ± 1.7% in this time (*p* = 0.0009). However, more signal was lost in the highest translation polysomes (mRNA bound by 4 or more ribosomes), indicating that absolute levels of translation were likely decreased by more than 20%. As a separate measure of translation rate, surface sensing of translation (SUnSET) was optimized for use under DR conditions in *C. elegans*. Previous application of SUnSET in *C. elegans* relied on ingestion of bacteria mixed with puromycin (Arnold et al., 2014; Heissenberger et al., 2020). To allow puromycin incorporation in worms without food (i.e., from fasted animals), the mutant strain *bus-5(br19)* was used due to its enhanced cuticle permeability (Xiong et al., 2017), allowing this strain to incorporate puromycin at a higher rate compared to N2 (Somers et al., 2022). In this assay, *bus-5(br19)* animals were incubated with puromycin for 1 h followed by Western blot to detect puromycin signal. N2 animals were included for comparison. As expected, since the 1 h incubation was much shorter than the traditional puromycin assay, almost no puromycin signal could be detected in N2 (Figure 1B). In contrast, the *bus-5(br19)* mutant showed clear puromycin incorporation in both AL and fasting conditions. However, almost no signal was observed in worms treated with cycloheximide, an inhibitor of mRNA translation. A reduction in translation by 37.3% was detected in worms fasted for 1 h and 48.9% after 3 h (Figure 1B, lower panel). Taken together, both types of translation assay demonstrate translation reduction under short-term FT. Furthermore, the modified SUnSET method utilizing *bus-5(br19)* mutants provides an effective approach for assessing translational changes in *C. elegans* that is not affected by differences in food availability or pharyngeal pumping rate of worms.

It is not clear what role ISR plays in the attenuated translation and extended longevity under DR conditions. We performed Western blot to detect the activation of the ISR by eIF-2α phosphorylation. We included the knockout mutants *gcn-2(ok871)* and *pek-1(ok275)*, which were crossed to generate the double mutant *gcn-2(ok871);pek-1(ok275)*. We also included a mutant *eif-2α(rog3)*, hitherto referred to simply as *eif-2α*, with serine 49 replaced by alanine, which cannot be phosphorylated. All these mutants and N2 were treated with AL or FT conditions for 3 h (Figure 1C). In N2, fasted animals showed higher eIF-2α phosphorylation compared to AL, indicating that the ISR was activated under the fasting condition. In the mutants, only *pek-1(ok275)* showed a similar pattern to wild type, whereas the signal could not be detected in other mutants. Results indicate that GCN-2, but not PEK-1, is responsible for eIF-2α phosphorylation under FT conditions as expected.

### Early changes in translation upon fasting are similar in WT and ISR mutants

**FIGURE 2:**
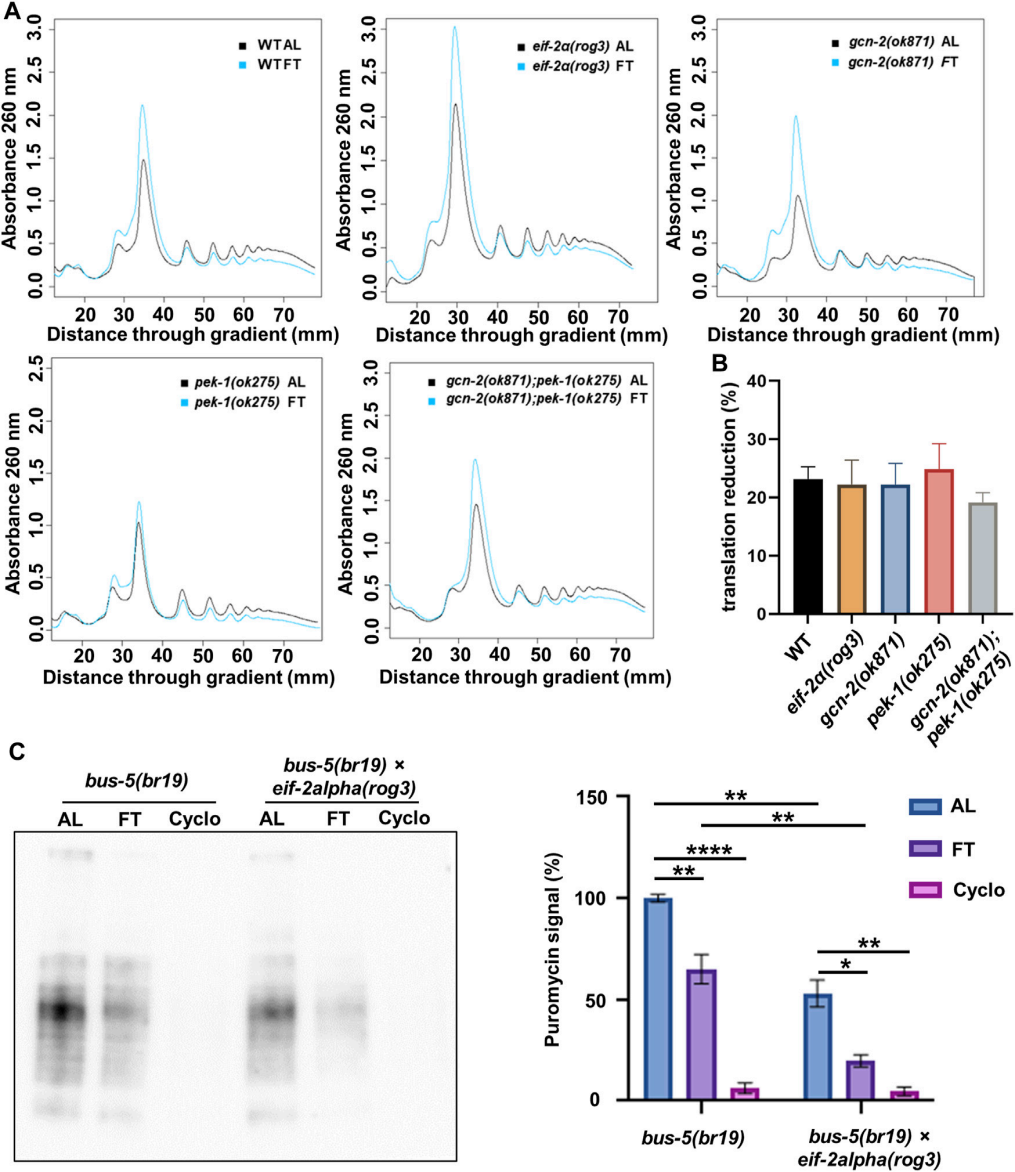
eIF-2α phosphorylation is not required for downregulation of translation upon withdrawal of food. **(A)** polysome profiling of day 1 adult N2, *eif-2α(rog3)*, *gcn-2(ok871), pek-1(ok275),* and *gcn-2(ok871);pek-1(ok275)* after *ad libitum* feeding (AL) or fasting (FT) for 3 h. Each profile is representative of 3 independent experiments. **(B)** Quantification of translation reduction (%). Error bars represent means ± SEM. One-way ANOVA was performed and no statistical difference was detected. **(C)** SUnSET puromycin assay using Western blot with anti-puromycin antibodies to detect translation change in *bus-5(br19)* and *bus-5(br19);eif-2α(rog3)* day 1 adult worms under 3 h AL, 3 h FT and 10 mM cycloheximide treatments. Representative membrane is shown. Quantification was conducted with 3 replicates. Error bars represent means ± SEM. One-way ANOVA with Dunnett’s *post hoc* test was performed (*p < 0.05; **p < 0.01; ***p < 0.001; ****p < 0.0001).

Next, we wanted to see how translation is affected in phosphorylation and kinase mutants under short-term fasting conditions. Therefore, we used polysome profiling and SUnSET to determine how translation is influenced in ISR mutants after 3 h FT. Polysome profiling showed that, regardless of the mutations, translational repression was observed in the *eif-2α* phospho-null mutant as well as *gcn-2(ok871)* and *pek-1(ok275)* (Figure 2A). In each instance, polysomes were reduced about 20% (Figure 2B), indicating that phosphorylation of eIF-2α is not required for these early changes in translation when food is removed.

In order to compare total changes in translation between control and the *eif-2α* phosphorylation mutant using SUnSET, *bus-5(br19)* was crossed with *eif-2α* mutants and animals were fasted for 3 h (Figure 2C). Both *bus-5(br19)* and *bus-5(br19);eif-2α* mutants showed reduced translation under FT conditions. Interestingly, translation is constitutively low in *eif-2α* phospho-null mutants compared to WT animals in both AL and fasting conditions (Figure 2C). Constitutively low translation is interesting from the point of view of NMD, which requires translation for its activity and which is, itself required for longevity associated with DR in *C. elegans* (Rollins et al., 2019; Kim et al., 2020). Importantly, results indicate that translation is downregulated in *eif-2α* phospho-mutants under FT compared to AL conditions (Figure 2C). Thus, the ISR is not required for translation attenuation under DR conditions.

### Translation-dependent changes in NMD driven by fasting do not require the ISR

**FIGURE 3:**
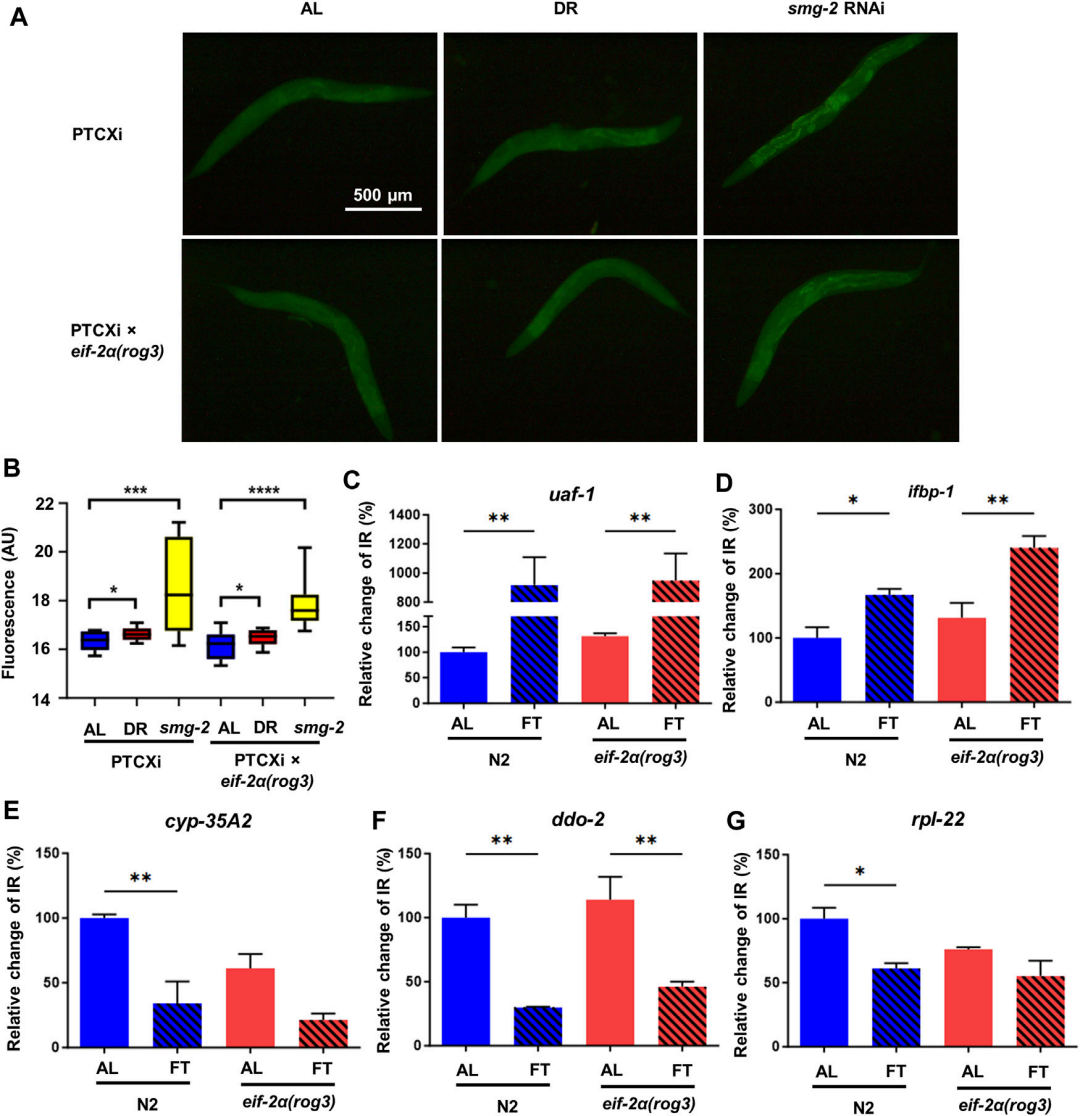
eIF-2α phosphorylation is dispensable for NMD under DR conditions. **(A)** Representative microscopy images of NMD reporter PTCXi and PTCXi crossed with *eif-2α(rog3)*. **(B)** GFP signal quantification shown in **(A)** from 3 experiments performed. For **(A)** and **(B)**, day 1 adult worms were placed under *ad libitum* feeding (AL), dietary restriction (DR) or *smg-2* RNAi for 3 days prior to imaging. **(C–G)** Intron retained (IR) isoform expression of *uaf-1* **(C)**, *ifbp-1* **(D)**, *cyp-35A2* **(E)**, *ddo-2* **(F)**, and *rpl-22* **(G)** in N2 and *eif-2α(rog3)* after 3 days of AL or fasting (FT) in young adult worms. IR expression was normalized by expression of all isoforms of each gene. Error bars represent means ± SEM (n = 3). One-way ANOVA with Dunnett’s *post hoc* test was performed. *p < 0.05; **p < 0.01; ***p < 0.001; and ****p < 0.0001.

The activation of NMD relies on translation, thus we sought to confirm whether the ISR downregulates NMD when food is removed. To test its activity, we used the NMD reporter strain PTCXi harboring a PTC that normally abrogates expression of a GFP reporter (Longman et al., 2007). In environments without stress where NMD is highly active, the PTC containing transcript is degraded by NMD, precluding expression of GFP. We tested wild type and the *eif-2α* phospho-deficient mutant in response to DR. *smg-2* RNAi was used as a control, since this gene is required for NMD. As expected, the worms on *smg-2* RNAi showed the highest GFP signal. The animals under DR also increased their fluorescence significantly compared to AL, but not as strong as the one in *smg-2* RNAi, indicating NMD decreased under DR, but did not turn off completely (Figures 3A, B). The fluorescence in the NMD reporter crossed with *eif-2α* presented a very similar pattern. The worms under DR increased GFP signal significantly, suggesting that NMD was also downregulated in the phospho-null mutant and that the ISR was not required for suppressed NMD activity under DR.

To better understand the relationship between ISR and NMD, we measured expression of certain transcripts with intron retention (IR), which causes out-of-frame translation and generates at least one PTC. We tested the isoform expression relative to productively spliced transcripts in N2 and *eif-2α* phospho-mutants under AL and FT conditions for 3 days. The first set of genes tested were the alternatively spliced ribosomal protein mRNA *rpl-1*, *rpl-3, rpl-7A*, and *rpl-12* that are known NMD targets (Mitrovich and Anderson, 2000). Except *rpl-7A* that did not show expression change, the IR containing isoforms of *rpl-1*, *rpl-3,* and *rpl-12* decreased in FT compared to AL significantly (Supplementary Figure S1). Results suggest that alternative splicing decreased and/or NMD increased for these factors. We also analyzed the expression of *atf-4*, which is the main ISR effector (Supplementary Figure S1E) but did not observe any statistical change in the expression level (*p* = 0.09 for N2 AL-DR comparison). Furthermore, the expression pattern was very consistent in wild type and *eif-2α* in all these genes, indicating that these large ribosomal subunit transcripts are not dependent on the ISR for changes in NMD. We further tested the expression of the same IR transcripts in *gcn-2(ok871);pek-1(ok275)* to confirm the relationship between ISR and NMD. Similarly, under FT only *uaf-1* and *ifbp-1* increased their expression, but not any other transcripts tested (Supplementary Figure S2 and Supplementary Figure S3), The same pattern was observed in the wild type, confirming again that ISR does not regulate NMD under FT.

We then analyzed the expression of IR containing mRNA with increased abundance under conditions of a food dilution form of DR according to RNAseq data from a previous study (Rollins et al., 2019). We selected the isoforms with IR of *uaf-1*, *ifbp-1*, *cyp-35A2*, *ddo-2*, and *rpl-22*. UAF-1 is a large subunit of splicing factor U2AF (U2 auxiliary factor) involved in the recognition of 3’ splice sites (Zorio and Blumenthal, 1999; Ma and Horvitz, 2009). IFBP-1 is an ortholog of human IRF2BP2 and IRF2BPL, which are members of the IRF2BP (interferon regulatory factor 2 binding protein) family of transcriptional repressors (Childs, 2003), while CYP-35A2 is a protein in Cytochrome P450 family (Lim et al., 2022). DDO-2 encodes D-aspartate oxidase that degrades D-amino acids (Saitoh et al., 2012). RPL-22 is a large subunit of ribosomal protein. In these mRNAs, *uaf-1* showed the highest increase of expression from 100% to 934.6% in N2 and 134.3% of the N2 AL value to 963.9% in *eif-2α* respectively (Figure 3C). The fact that UAF-1 is a splicing factor highlights the self-regulation of splicing factors through Alternative splicing and NMD (AS-NMD) (Lejeune, 2022) The alternatively spliced isoform of *ifbp-1* also went up 81.9% in N2 and 72.55% in *eif-2α* respectively (Figure 3D). The higher levels of *uaf-1* and *ifbp-1* with IR under FT conditions is suggestive of downregulation of NMD for these genes. Interestingly, *cyp-35A2, ddo-2, and rpl-22* that increased IR events under DR as previously reported did not increase their abundance in fasted animals (Figures 3E–G), which could be due to experimental condition variation (i.e., food dilution versus FT). However, the gene expression pattern in all these genes were very similar between N2 and *eif-2α*, suggesting again that the ISR is not a key player to regulate NMD when nutrients are limited.

### The ISR is dispensable for increased lifespan under DR

**FIGURE 4:**
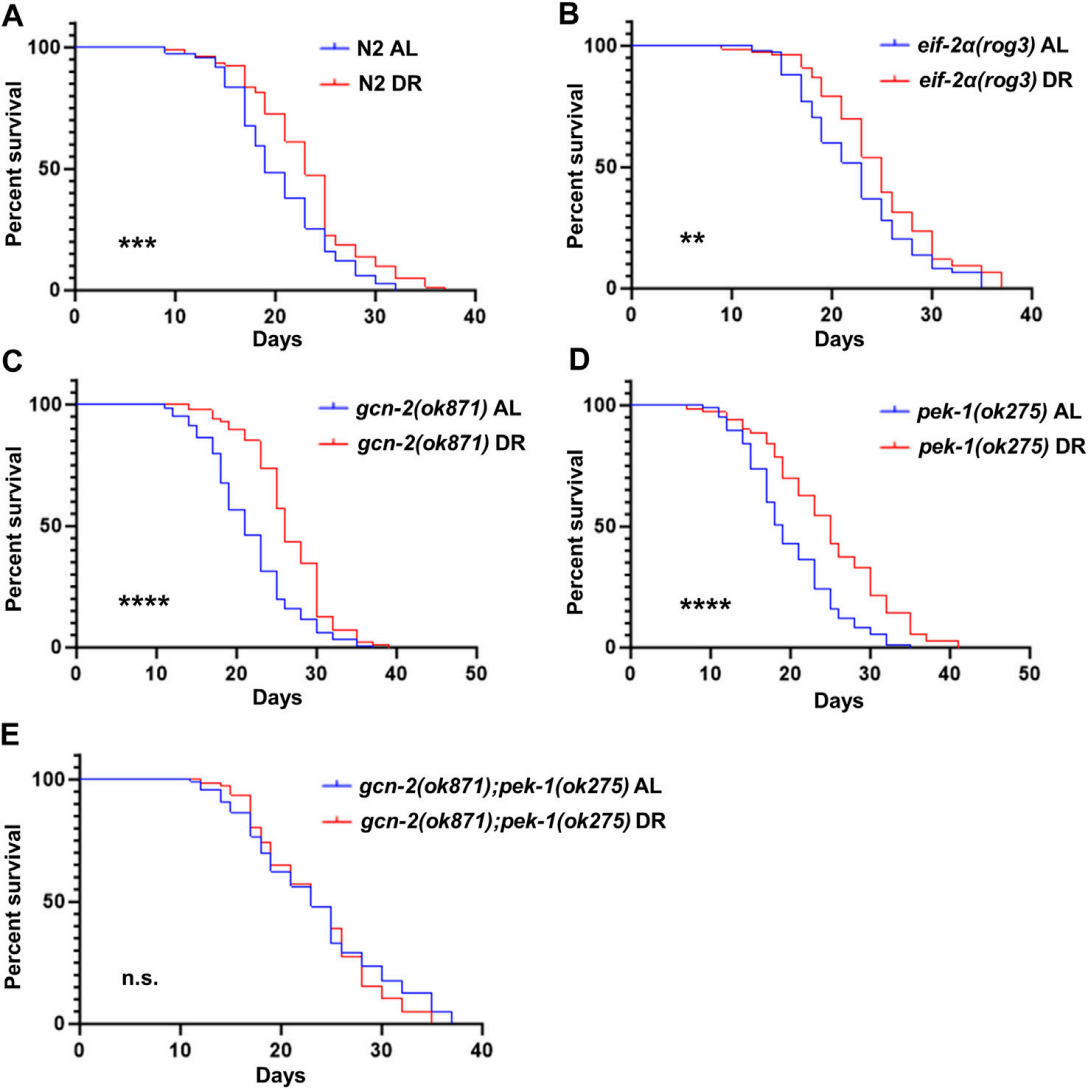
eIF-2α phosphorylation is not required for increased survival under DR. Worms were fed AL or DR diets starting from day 1 of adulthood. Percent survival was plotted for **(A)** N2, **(B)** *eif-2α(rog3)*, **(C)** *gcn-2(ok871),* **(D)** *pek-1(ok275)*, and **(E)** *gcn-2(ok871);pek-1(ok275)*. Kaplan–Meier survival curves were compared using the Mantel–Cox log-rank test. *p < 0.05; **p < 0.01; ***p < 0.001; and ****p < 0.0001. See Supplementary Table S3 for additional details and replicates.

To address whether inactivation of the ISR displays detrimental effects on longevity under DR, we compared lifespan of *eif-2α* phosphorylation and ISR kinase mutants under AL and DR. Similar to N2, DR promoted lifespan in *eif-2α, gcn-2(ok871),* and *pek-1(ok275)*, indicating that neither phosphorylation of eIF-2α, nor the individual kinases targeting this translation factor, are needed for lifespan extension under DR (Figure 4; Supplementary Table S3). Interestingly, the lifespan of double mutant *gcn-2(ok871);pek-1(ok275)* under DR was not significantly different from AL (Figure 4E). This observation suggests more than one phosphorylation target is regulated by these kinases. As with eIF-2α, the target may be shared between GCN-2 and PEK-1. Taken together with earlier results, the ISR is not required for changes in translation, NMD, or lifespan associated with DR. Furthermore, *eif-2α* lived longer than wild type under AL feeding (*p* = 0.0053; Supplementary Figure S4 and Supplementary Table S3), indicating that the inability to activate the ISR positively influences longevity under the conditions tested. This finding is consistent with previously reported results (Derisbourg et al., 2021b).

### The ISR is required for protection from ER stress, but not for fasting or thermal stress

**FIGURE 5:**
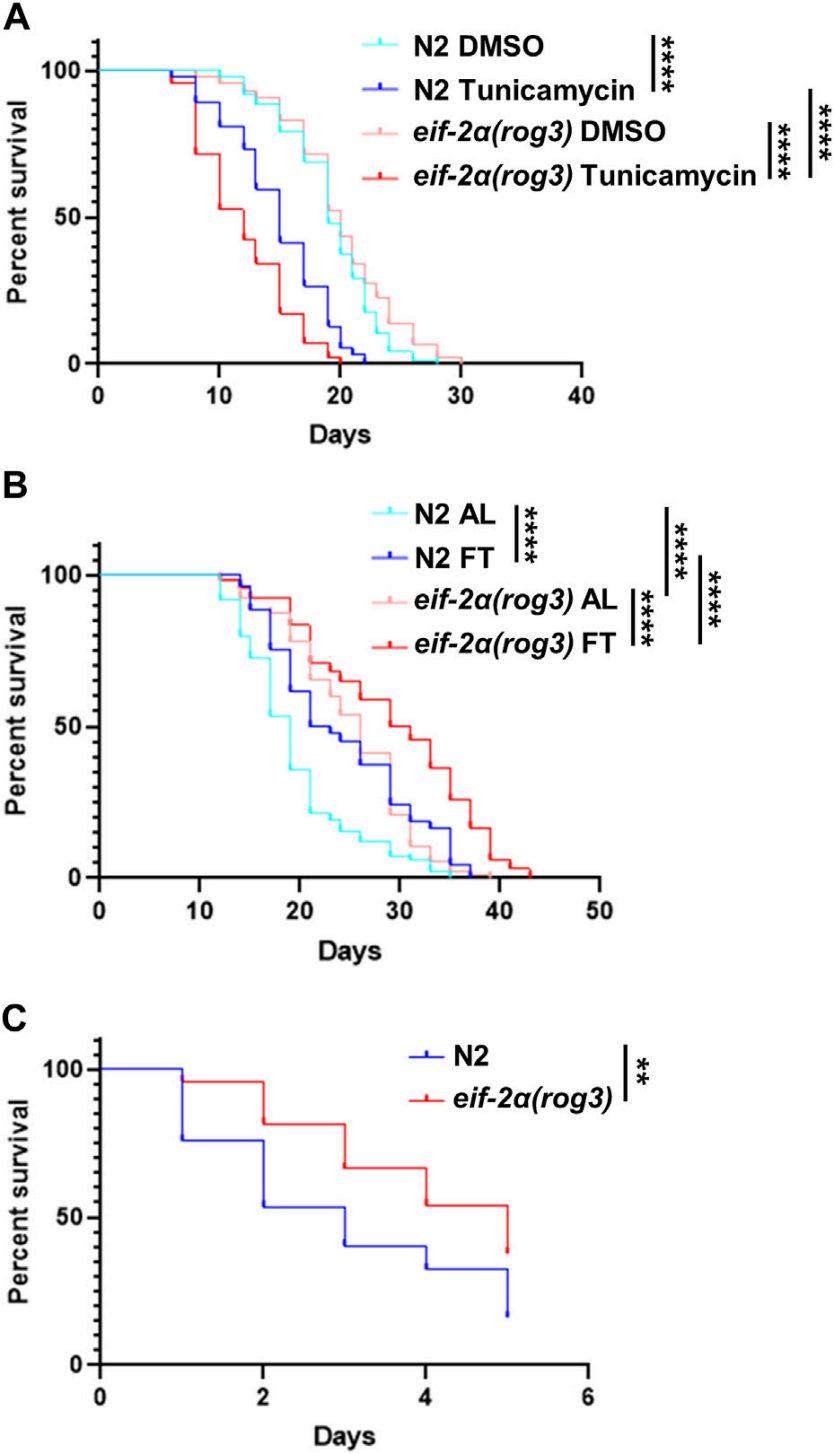
eIF-2α phosphorylation is required for normal survival under ER stress, but not for thermal stress. **(A)** Survival of worms in DMSO (control) or 25 μg/mL tunicamycin starting from day 1 adulthood. **(B)** Lifespan of worms under AL or fasting (FT) treatments starting from day 1 adulthood. **(C)** Heat stress recovery assay. Worms were incubated at 35°C for 4 h at day 7 of adulthood then shifted to 20°C. The survival was scored for 5 days continuously. Kaplan–Meier survival curves were compared using the Mantel–Cox log-rank test. *p < 0.05; **p < 0.01; ***p < 0.001; and ****p < 0.0001. See Supplementary Tables S4–S6 for additional details and replicates.

Considering that the ISR is dispensable under DR involving food dilution, we wanted to see if it is required for other forms of stress. To induce ER stress, *C. elegans* were treated continuously with tunicamycin starting from young adulthood. Compared to N2, the survival of *eif-2α* was reduced significantly under ER stress (Figure 5A; Supplementary Table S6). Thus, the ISR is required for a wild-type level of survival under conditions of ER stress.

Given the lack of requirement for the ISR in lifespan extension under DR (Figure 4), we wondered if imposing a stronger form of DR requires the ISR for enhanced survival, since this may lead to a greater level of uncharged tRNA associated with its activation. Under fasting conditions starting from young adulthood, both N2 and *eif-2α* exhibited an extension in lifespan characteristic of this form of DR (Figure 5B; Supplementary Table S5). Notably, under both AL and fasting conditions, the mutant demonstrated a longer lifespan compared to wild-type animals. We also tested the lifespan of *gcn-2(ok871);pek-1(ok275)* under fasting due to its non-extended lifespan under DR (Supplementary Figure S5). The results were similar to the one under DR. The survival rate of the double mutant did not differ from AL, also suggesting an unidentified shared target exists between GCN-2 and PEK-1 essential for lifespan extension in nutrient deprivation.

Although heat stress is not typically associated with the ISR, we included it since increased resistance to heat stress is frequently associated with gene changes that increase lifespan as observed for the *eif-2α* mutant under food dilution DR (Figure 4B and Supplementary Table S3) and fasting (Figure 5B). To investigate whether the ISR is involved in the response to heat stress, day 7 adult worms were incubated at 35°C for 4 h and their survival was scored daily thereafter. The inhibition of EIF-2α phosphorylation did not decrease the survival rate after heat stress (Figure 5C; Supplementary Table S4). As observed with survival under standard conditions, the *eif-2α* phospho-null mutant showed increased survival compared to N2.

### Extended lifespan in eIF-2α phospho-null mutants is associated with decreased fecundity

**FIGURE 6:**
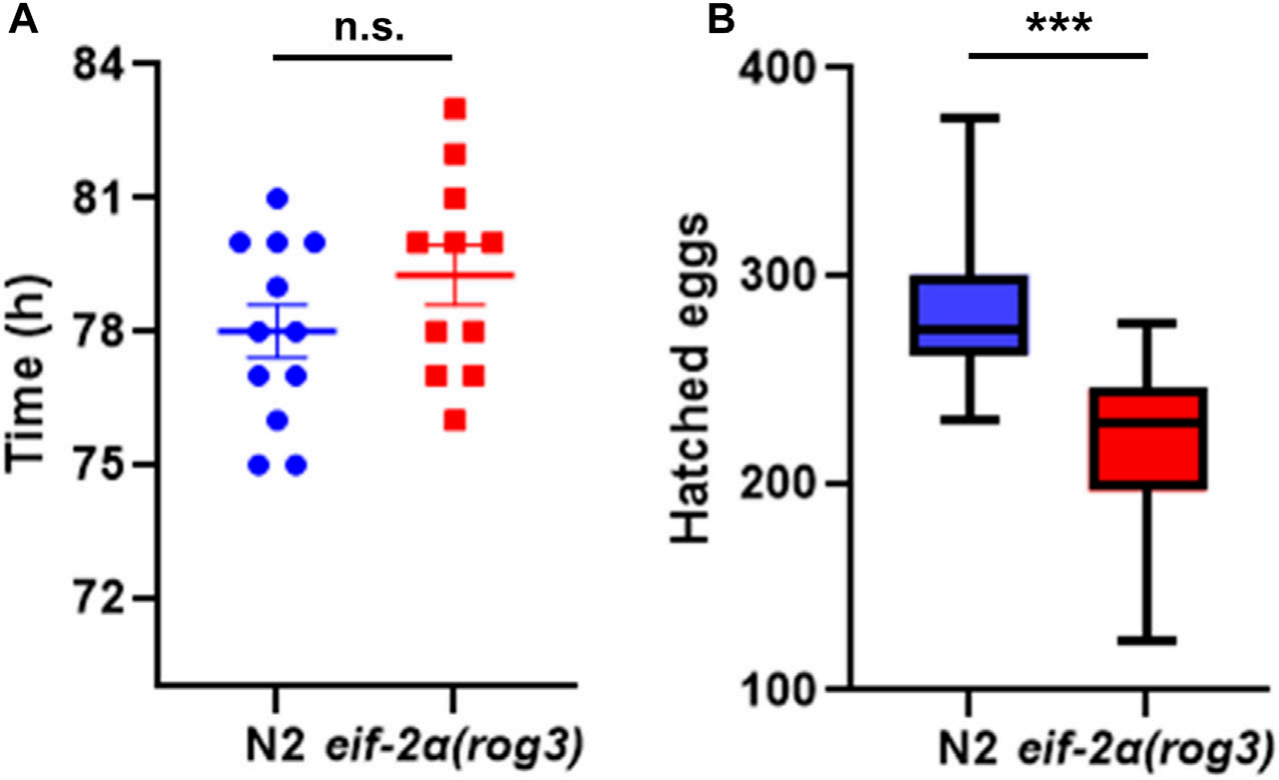
eIF-2α phospho-null mutant has smaller brood size. **(A)** Development time and **(B)** hatched egg numbers of N2 and *eif-2α(rog3)*. Representative results of 3 independent experiments are shown. In each experiment, at least 12 worms of each strain were included. Error bars represent means ± SEM. Student’s t-test was performed to determine significance. *p < 0.05; **p < 0.01; ***p < 0.001; and ****p < 0.0001.

Since *eif-2α* lived longer than N2 animals, we investigated if ISR inhibition affects development and reproduction of animals. We measured the development time of *eif-2α* and found that it was not significantly different between the mutant and N2, with both taking between 78 and 80 h to go from egg to egg-laying adult (Figure 6A). However, the total number of progeny was significantly lower in *eif-2α* compared to N2 (Figure 6B). Therefore, inhibition of the ISR leads to an extension of lifespan in the absence of stress, but it also showed diminished reproductive ability. This is consistent with trade-offs frequently observed in long-lived mutants.

### The ISR is only activated by PEK-1 in response to ER stress

**FIGURE 7:**
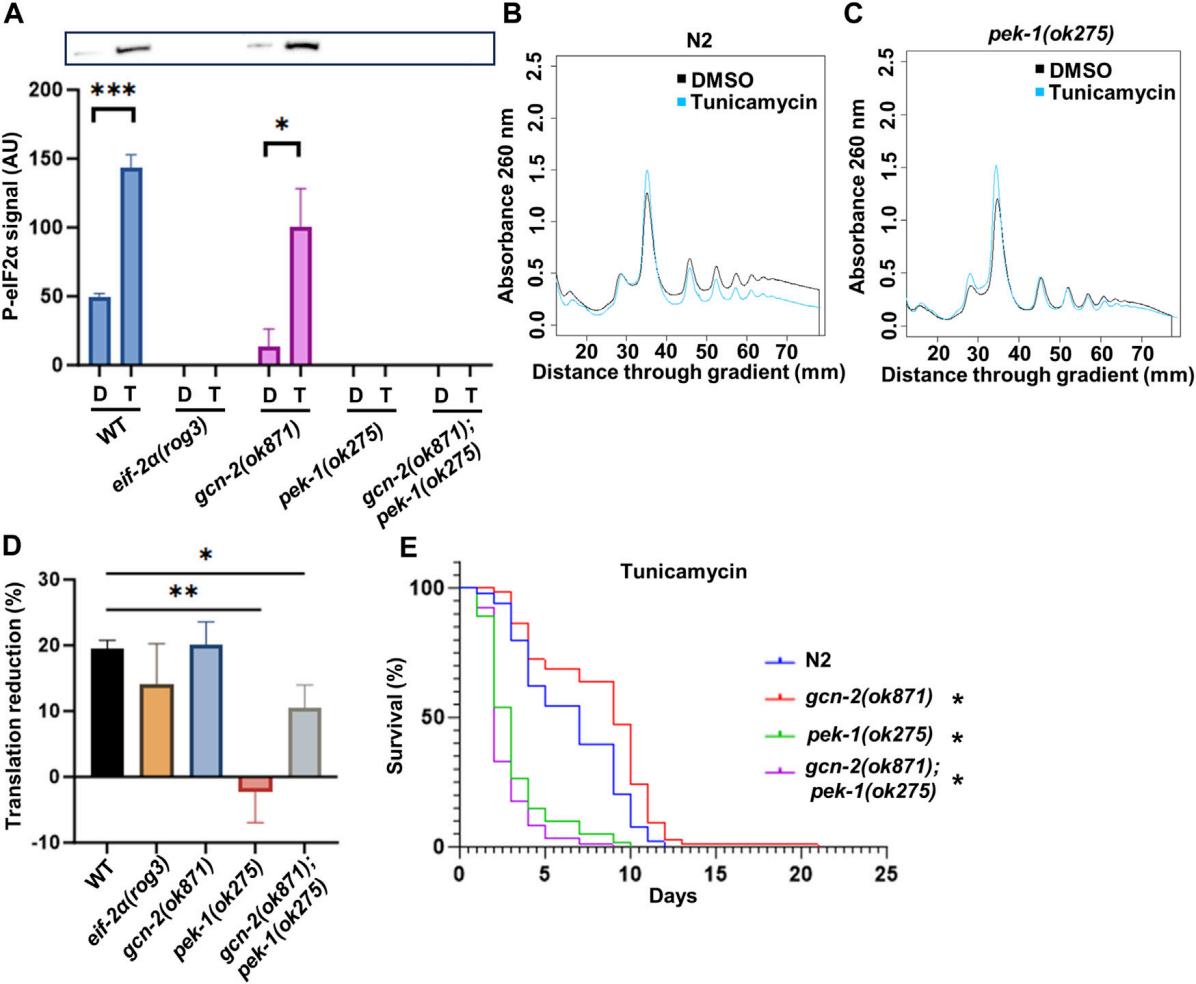
The importance of eIF-2α phosphorylation under ER stress. **(A)** Western blot with anti-eIF2α phosphorylation antibody. Representative membrane is shown. **(B,C)** Polysome profiling of day 1 adult N2 **(B)** and *pek-1(ok871)* **(C)**. Day 1 adult worms were treated with DMSO or 25 μg/mL tunicamycin for 3 h prior to cell lysis. **(D)** Quantification of translation reduction. Error bars represent means ± SEM (n = 3). One-way ANOVA with Dunnett’s *post hoc* test was performed. See Supplementary Figure S6 for the polysome profiling of the other strains. **(E)** Survival of worms in 25 μg/mL tunicamycin starting from day 1 adulthood. See Supplementary Table S7 for additional details and replicates. Kaplan–Meier survival curves were compared using the Mantel–Cox log-rank test. *p < 0.05; **p < 0.01; ***p < 0.001; and ****p < 0.0001.

To gain further insights into the response of the ISR to ER stress, we tested the eIF-2α phosphorylation status of kinase mutants upon exposure to tunicamycin. After 3 h of tunicamycin exposure, only N2 and the *gcn-2(ok871)* mutant were able to phosphorylate eIF2α, as expected. Conversely, *pek-1(ok275)* and *gcn-2(ok871);pek-1(ok275)* double mutants could not (Figure 7A), showing that only PEK-1 is responsible for ISR activation under this condition.

Since PEK-1 is required for normal survival and eIF-2α phosphorylation in response to tunicamycin, we wondered whether translational downregulation associated with ER stress and ISR activation was impaired in *pek-1* mutants. Polysome profiling was conducted after the worms were challenged with tunicamycin for 3 h. In N2, the polysome fraction was reduced by 19.5% (Figures 7B, D). In the *pek-1* mutant, translation remained near DMSO control levels in the presence of tunicamycin (Figures 7C, D), which is consistent with the fact that *pek-1* is essential to activate ISR under ER stress. The double mutant *gcn-2(ok871);pek-1(ok275)* also exhibited a muted response compared to N2 (Figure 7D and Supplementary Figure S6C). There was no statistical difference between *pek-1(ok275)* and the double mutant (*p* = 0.093). Surprisingly, the *eif-2α* phospho-mutant showed similar reduction in translation to N2 (Figure 7D and Supplementary Figure S6A), despite the importance of this residue for survival under ER stress (Figure 5A). To confirm the importance of PEK-1 for survival under ER stress, we challenged the ISR kinase mutants with long-term tunicamycin exposure. As expected, the survival rate of *pek-1(ok275)* and *gcn-2(ok871);pek-1(ok275)* dropped dramatically (Figure 7E) while the DMSO control worms all showed similar lifespans (Supplementary Figure S6D). Taken together, PEK-1 activates the ISR under ER stress and the ISR is necessary for the ER stress response.

## Discussion

In this study, we explored the role of the ISR in various stress conditions. We found that the ISR plays a critical role in the ER stress response. Specifically, eIF2α was phosphorylated by PEK-1 under ER stress, and inhibition of ISR activation not only hindered attenuation of total translation but also showed detrimental effects on survival. Conversely, the ISR was not required for lifespan extension under food dilution or fasting conditions. Despite an increase in GCN-2 mediated eIF-2α phosphorylation during fasting, the ISR was not required for translation suppression in *gcn-2* or *eif-2α* phospho-mutants. Furthermore, ISR inhibition did not impact the ability to differentially regulate NMD according to levels of intron-bearing mRNA of several genes tested. Interestingly, inhibiting phosphorylation of eIF2α at S49 via S-to-A mutation extended lifespan in the absence of stress. However, this came at the cost of reduced fecundity, indicating a trade-off between lifespan extension and reproductive ability.

Monitoring translation changes that suppress and redirect protein synthesis is critical to understand stress response mechanisms. Low translation states are frequently associated with increased lifespan. Consequently, the availability of reliable methods to measure translation within specific time frames and under particular treatments is critical to comprehending the underlying genetic mechanisms. By accurately measuring translation changes, researchers can assess the impact of specific stress or treatments, and further unravel the complex interplay between genetic regulation and stress responses. Various approaches have been developed to measure translation quantitatively. One common method is polysome profiling, which allows for isolation of actively translated mRNA and separates ribosomal subunits, monosomes, and polysomes (Merret et al., 2015; Chassé et al., 2017; Rollins et al., 2019). This type of profiling measures not just total translation but can also be used to measure gene-specific translation efficiency according to expression of specific mRNA within isolated polysomes. However, polysomal analysis is time consuming and requires a large quantity of worms to generate profiles. Therefore, it is advantageous to have an alternative method to quantify translation. The SUnSET puromycin assay is a fitting solution in this regard. Puromycin replaces aa-tRNA to enter the ribosome, causing translation termination and subsequent release of puromycin-bound peptidyl-tRNA. The levels of puromycylation directly correlate with the overall rate of translation (Aviner, 2020). Thus, its incorporation enables the quantification of global translation. Quantification can be achieved biochemically via Western blotting or by imaging fluorescently labeled puromycin in individual worms (Somers et al., 2022). In this study, we further refined the assay by optimizing the protocol using the *bus-5(br19)* mutant and short-period puromycin incubation. In this way, incorporation does not rely on food source, making it ideal to measure translation even under fasting conditions.

The phosphorylation of eIF2α can be uncoupled from downstream ISR effectors (Wanders et al., 2016; Pettit et al., 2017). While activated, the ISR attenuates global protein synthesis and promotes the translation of specific mRNAs. The four kinases GCN2, PERK, PKR, and HRI phosphorylate eIF2α under different stress conditions. Each of these kinases bear conserved kinase and regulatory domains allowing them to respond to diverse conditions (Costa-Mattioli and Walter, 2020). In nutrient restriction stress, studies reported increased levels of eIF2α phosphorylation and ATF4 expression (Lee et al., 2008; Sikalidis and Stipanuk, 2010; Sikalidis et al., 2011). However, eIF2α phosphorylation does not always correlate with ATF4 expression and overall protein changes. Feeding a methionine-restricted diet to wild-type and *Gcn2* ^*−/−*^ mice had similar effects on many genes regulated by the ISR, including ATF4 (Pettit et al., 2017). Similarly, methionine restricted diet was reported to activate the ISR (Rajanala et al., 2019) and results in phenotypes (Wanders et al., 2016) that are independent from GCN2 and PERK. In our study, the role of *C. elegans* ATF-4 was not directly assessed, although its expression was not significantly changed under DR in either N2 wild type or *eif-2α* phospho-null mutants. Indeed, although GCN-2 activates the ISR in *C. elegans* during fasting (Figure 1C), it is not required to downregulate translation or to increase lifespan under nutrient-limiting conditions (Figures 2, 4).

ISR kinases harbor functions beyond eIF-2α phosphorylation. PERK not only phosphorylates eIF2α to induce global attenuation of protein synthesis (Harding et al., 2000) and stimulate expression of stress response proteins, but is also involved in activation of ATF6, which increases expression of UPR target genes in mice (Teske et al., 2011). Our findings are in line with the function of PERK across various species, particularly in regard to translational downregulation and survival under ER stress. Interestingly, one study found that, while the phosphorylation of eIF2α was reduced in PERK^−/−^ fibroblasts under ER stress, GCN2 was able to compensate partially for the loss of PERK (Hamanaka et al., 2005). In our study, GCN-2 was only active under FT and PEK-1 was only active under ER stress with respect to eIF-2α phosphorylation (Figures 1C, 7A). This does not necessarily mean that GCN-2 and PEK-1 cannot compensate for each other’s activity. For example, the compensation may target a different phosphorylation site on eIF-2α besides S49. The notion that other kinase targets may exist is supported by results from the *gcn-2(ok871);pek-1(ok275)* double mutant, which did not show lifespan extension under DR whereas the single kinase and eIF-2α phospho-mutants did (Figure 4). Additionally, the translation reduction was completely inhibited in the loss-of-function *pek-1* mutant, but not in the *eif-2α* phospho*-*mutant (Figure 7D). Both observations suggest that eIF2α may have other important phosphosites related to ISR activation or some other targets of GCN-2 or PEK-1 may exist. For example, Cullinan et al. showed that PERK also phosphorylates Nrf2 transcription factor (SKN-1 in *C. elegans*) and stimulates dissociation of the cytoplasmic Nrf2/Keap1 complex (Cullinan et al., 2003). Nrf2 deletion decreased cell survival rate compared to wild type after inducing ER stress (Cullinan et al., 2003). To our knowledge, Nrf2 is the only target of PERK besides eIF2α that has been identified.

As previously reported, the ISR and NMD coregulate each other in response to stress. Both hypoxia and ER stress suppress NMD via eIF2α phosphorylation in mammalian cell lines (Gardner, 2008; Usuki et al., 2013) and the ISR factor ATF4 is otherwise subject to degradation by NMD. Conversely, it was reported recently that NMD inhibition via pharmacologic disruption of SMG1 activated the ISR in mice, revealing a bidirectional relationship. (De La Peña et al., 2023). However, in our study, eIF2α phosphorylation was not required for NMD changes during fasting (Figure 3, Supplementary Figure S1). Whether NMD inactivation affects ISR activity was not examined and will need further exploration. Under DR, NMD is downregulated but not completely inactive (Figure 3). In fact, NMD still plays an important role in specific gene expression regulation, since loss of NMD factors negatively affects benefits of increased longevity under DR (Rollins et al., 2019; Kim et al., 2020).

AS-NMD is usually coupled to regulate expression of specific genes to adapt to environmental stress. One study showed that RNP-6 modulates alternative splicing events and regulates longevity by mTORC1 downregulation (Huang et al., 2022). Similarly, under DR, as a result of AS-NMD, IR events increased in both translated and total fraction showing specific gene expression regulation (Rollins et al., 2019). The existence of PTC-containing transcripts among polysomes suggests that a higher proportion of these transcripts evaded the surveillance by NMD. Most of PTC-containing transcripts remain unfunctional, but a few of them can be translated and synthesize different protein variants (Ge and Porse, 2014). In this study, we noticed that IR events increased for specific genes under fasting, specifically for those encoding the splicing factor UAF-1 and transcriptional regulator IFBP-1 (Figures 3C,D). IR favors producing more varieties of protein isoforms, which is critical for restoring transcriptome homeostasis in response to stress. Some of the intron-retained isoforms decreased under FT in our experiments (e.g., *cyp-35A2, ddo-2* and *rpl-22;* Figures 3E–G). This can be due to two reasons. One is that under FT the total translation activity goes down causing the lower transcription activity of these isoforms. Another is that even though NMD is downregulated in nutrient scarcity, it is still critical for specific gene expression regulation. Thus, NMD may further degrade the IR isoforms of these genes.

This study explored the role of ISR, especially eIF-2α phosphorylation, under stress and DR conditions. Further studies are needed to look into the potential for additional targets of GCN-2 and PEK-1, as well as the role AS-NMD plays in changing mRNA processing to differentially regulate gene expression under nutrient limiting conditions.

## Data Availability

The original contributions presented in the study are included in the article/Supplementary Material, further inquiries can be directed to the corresponding author.

## References

[B1] Arnold A. Rahman M. M. Lee M. C. Muehlhaeusser S. Katic I. Gaidatzis D. (2014). Functional characterization of *C. elegans* Y-box-binding proteins reveals tissue-specific functions and a critical role in the formation of polysomes. Nucleic Acids Res. 42, 13353–13369. 10.1093/nar/gku1077 25378320 PMC4245946

[B2] Aviner R. (2020). The science of puromycin: from studies of ribosome function to applications in biotechnology. Comput. Struct. Biotechnol. J. 18, 1074–1083. 10.1016/j.csbj.2020.04.014 32435426 PMC7229235

[B3] Baker B. M. Nargund A. M. Sun T. Haynes C. M. (2012). Protective coupling of mitochondrial function and protein synthesis via the eIF2α kinase GCN-2. PLOS Genet. 8, e1002760. 10.1371/journal.pgen.1002760 22719267 PMC3375257

[B4] Barber G. N. (2001). Host defense, viruses and apoptosis. Cell Death Differ. 8, 113–126. 10.1038/sj.cdd.4400823 11313713

[B5] Bond S. Lopez-Lloreda C. Gannon P. J. Akay-Espinoza C. Jordan-Sciutto K. L. (2020). The integrated stress response and phosphorylated eukaryotic initiation factor 2α in neurodegeneration. J. Neuropathology Exp. Neurology 79, 123–143. 10.1093/jnen/nlz129 PMC6970450 31913484

[B6] Bunpo P. Cundiff J. K. Reinert R. B. Wek R. C. Aldrich C. J. Anthony T. G. (2010). The eIF2 kinase GCN2 is essential for the murine immune system to adapt to amino acid deprivation by asparaginase. J. Nutr. 140, 2020–2027. 10.3945/jn.110.129197 20861212 PMC2955878

[B7] Butcher S. K. Lord J. M. (2004). Stress responses and innate immunity: aging as a contributory factor. Aging Cell 3, 151–160. 10.1111/j.1474-9728.2004.00103.x 15268748

[B8] Chassé H. Boulben S. Costache V. Cormier P. Morales J. (2017). Analysis of translation using polysome profiling. Nucleic Acids Res. 45, e15. 10.1093/nar/gkw907 28180329 PMC5388431

[B9] Chen J.-J. (2014). Translational control by heme-regulated eIF2α kinase during erythropoiesis. Curr. Opin. Hematol. 21, 172–178. 10.1097/MOH.0000000000000030 24714526 PMC4124034

[B10] Childs K. S. Goodbourn S. (2003). Identification of novel co-repressor molecules for interferon regulatory factor-2. Nucleic Acids Res. 31, 3016–3026. 10.1093/nar/gkg431 12799427 PMC162335

[B11] Costa-Mattioli M. Walter P. (2020). The integrated stress response: from mechanism to disease. Science 368, eaat5314. 10.1126/science.aat5314 32327570 PMC8997189

[B12] Cullinan S. B. Zhang D. Hannink M. Arvisais E. Kaufman R. J. Diehl J. A. (2003). Nrf2 is a direct PERK substrate and effector of PERK-dependent cell survival. Mol. Cell. Biol. 23, 7198–7209. 10.1128/MCB.23.20.7198-7209.2003 14517290 PMC230321

[B13] De La Peña J. B. Chase R. Kunder N. Smith P. R. Lou T.-F. Stanowick A. (2023). Inhibition of nonsense-mediated decay induces nociceptive sensitization through activation of the integrated stress response. J. Neurosci. 43, 2921–2933. 10.1523/JNEUROSCI.1604-22.2023 36894318 PMC10124962

[B14] Derisbourg M. J. Hartman M. D. Denzel M. S. (2021a). Perspective: modulating the integrated stress response to slow aging and ameliorate age-related pathology. Nat. Aging 1, 760–768. 10.1038/s43587-021-00112-9 35146440 PMC7612338

[B15] Derisbourg M. J. Wester L. E. Baddi R. Denzel M. S. (2021b). Mutagenesis screen uncovers lifespan extension through integrated stress response inhibition without reduced mRNA translation. Nat. Commun. 12, 1678. 10.1038/s41467-021-21743-x 33723245 PMC7960713

[B16] Ecker N. Mor A. Journo D. Abeliovich H. (2010). Induction of autophagic flux by amino acid deprivation is distinct from nitrogen starvation-induced macroautophagy. Autophagy 6, 879–890. 10.4161/auto.6.7.12753 20647741

[B17] Gardner L. B. (2008). Hypoxic inhibition of nonsense-mediated RNA decay regulates gene expression and the integrated stress response. Mol. Cell. Biol. 28, 3729–3741. 10.1128/MCB.02284-07 18362164 PMC2423288

[B18] Ge Y. Porse B. T. (2014). The functional consequences of intron retention: alternative splicing coupled to NMD as a regulator of gene expression. BioEssays 36, 236–243. 10.1002/bies.201300156 24352796

[B19] Greer E. L. Brunet A. (2009). Different dietary restriction regimens extend lifespan by both independent and overlapping genetic pathways in *C. elegans* . Aging Cell 8, 113–127. 10.1111/j.1474-9726.2009.00459.x 19239417 PMC2680339

[B20] Haigis M. C. Yankner B. A. (2010). The aging stress response. Mol. Cell 40, 333–344. 10.1016/j.molcel.2010.10.002 20965426 PMC2987618

[B21] Hamanaka R. B. Bennett B. S. Cullinan S. B. Diehl J. A. (2005). PERK and GCN2 contribute to eIF2alpha phosphorylation and cell cycle arrest after activation of the unfolded protein response pathway. MBoC 16, 5493–5501. 10.1091/mbc.e05-03-0268 16176978 PMC1289396

[B22] Harding H. P. Novoa I. Zhang Y. Zeng H. Wek R. Schapira M. (2000). Regulated translation initiation controls stress-induced gene expression in mammalian cells. Mol. Cell 6, 1099–1108. 10.1016/S1097-2765(00)00108-8 11106749

[B23] Harding H. P. Zeng H. Zhang Y. Jungries R. Chung P. Plesken H. (2001). Diabetes mellitus and exocrine pancreatic dysfunction in Perk−/− mice reveals a role for translational control in secretory cell survival. Mol. Cell 7, 1153–1163. 10.1016/S1097-2765(01)00264-7 11430819

[B24] Heissenberger C. Rollins J. A. Krammer T. L. Nagelreiter F. Stocker I. Wacheul L. (2020). The ribosomal RNA m5C methyltransferase NSUN-1 modulates healthspan and oogenesis in *Caenorhabditis elegans* . eLife 9, e56205. 10.7554/eLife.56205 33289480 PMC7746234

[B25] Horn M. Denzel S. I. Srinivasan B. Allmeroth K. Schiffer I. Karthikaisamy V. (2020). Hexosamine pathway activation improves protein homeostasis through the integrated stress response. iScience 23, 100887. 10.1016/j.isci.2020.100887 32086012 PMC7033349

[B26] Howard A. C. Rollins J. Snow S. Castor S. Rogers A. N. (2016). Reducing translation through eIF4G/IFG-1 improves survival under ER stress that depends on heat shock factor HSF-1 in *Caenorhabditis elegans* . Aging Cell 15, 1027–1038. 10.1111/acel.12516 27538368 PMC5114698

[B27] Huang W. Kew C. Fernandes S. de A. Löhrke A. Han L. Demetriades C. (2022). Decreased spliceosome fidelity and egl-8 intron retention inhibit mTORC1 signaling to promote longevity. Nat. Aging 2, 796–808. 10.1038/s43587-022-00275-z 37118503 PMC10154236

[B28] Hug N. Longman D. Cáceres J. F. (2016). Mechanism and regulation of the nonsense-mediated decay pathway. Nucl. Acids Res. 44, 1483–1495. 10.1093/nar/gkw010 26773057 PMC4770240

[B29] Jackson R. J. Hellen C. U. T. Pestova T. V. (2010). The mechanism of eukaryotic translation initiation and principles of its regulation. Nat. Rev. Mol. Cell Biol. 11, 113–127. 10.1038/nrm2838 20094052 PMC4461372

[B30] Jiang J. C. Jaruga E. Repnevskaya M. V. Jazwinski S. M. (2000). An intervention resembling caloric restriction prolongs life span and retards aging in yeast. FASEB J. 14, 2135–2137. 10.1096/fj.00-0242fje 11024000

[B31] Jonsson W. O. Margolies N. S. Anthony T. G. (2019). Dietary sulfur amino acid restriction and the integrated stress response: mechanistic insights. Nutrients 11, 1349. 10.3390/nu11061349 31208042 PMC6627990

[B32] Kapahi P. Chen D. Rogers A. N. Katewa S. D. Li P. W.-L. Thomas E. L. (2010). With tor, less is more: a key role for the conserved nutrient-sensing tor pathway in aging. Cell Metab. 11, 453–465. 10.1016/j.cmet.2010.05.001 20519118 PMC2885591

[B33] Karam R. Lou C.-H. Kroeger H. Huang L. Lin J. H. Wilkinson M. F. (2015). The unfolded protein response is shaped by the NMD pathway. EMBO Rep. 16, 599–609. 10.15252/embr.201439696 25807986 PMC4428047

[B34] Kaya A. Lee B. C. Gladyshev V. N. (2015). Regulation of protein function by reversible methionine oxidation and the role of selenoprotein MsrB1. Antioxidants Redox Signal. 23, 814–822. 10.1089/ars.2015.6385 PMC4589106 26181576

[B35] Kim E. J. E. Son H. G. Park H.-E. H. Jung Y. Kwon S. Lee S.-J. V. (2020). *Caenorhabditis elegans* algn-2 is critical for longevity conferred by enhanced nonsense-mediated mRNA decay. iScience 23, 101713. 10.1016/j.isci.2020.101713 33225240 PMC7662852

[B36] Kim S. Kim D. K. Jeong S. Lee J. (2022). The common cellular events in the neurodegenerative diseases and the associated role of endoplasmic reticulum stress. Int. J. Mol. Sci. 23, 5894. 10.3390/ijms23115894 35682574 PMC9180188

[B37] Korennykh A. Walter P. (2012). Structural basis of the unfolded protein response. Annu. Rev. Cell Dev. Biol. 28, 251–277. 10.1146/annurev-cellbio-101011-155826 23057742

[B38] Kulalert W. Kim D. H. (2013). The unfolded protein response in a pair of sensory neurons promotes entry of *C. elegans* into dauer diapause. Curr. Biol. 23, 2540–2545. 10.1016/j.cub.2013.10.058 24316205 PMC3870035

[B39] Kyriakakis E. Princz A. Tavernarakis N. (2015). “Stress responses during ageing: molecular pathways regulating protein homeostasis,” in Stress responses. Editor Oslowski C. M. (New York, NY: Springer New York), 215–234. Available at: http://link.springer.com/10.1007/978-1-4939-2522-3_16 (Accessed March 26, 2015). 10.1007/978-1-4939-2522-3_16 25804759

[B40] Lee J.-I. Dominy J. E. Sikalidis A. K. Hirschberger L. L. Wang W. Stipanuk M. H. (2008). HepG2/C3A cells respond to cysteine deprivation by induction of the amino acid deprivation/integrated stress response pathway. Physiol. Genomics 33, 218–229. 10.1152/physiolgenomics.00263.2007 18285520

[B41] Lejeune F. (2022). Nonsense-mediated mRNA decay, a finely regulated mechanism. Biomedicines 10, 141. 10.3390/biomedicines10010141 35052820 PMC8773229

[B42] Li Z. Vuong J. K. Zhang M. Stork C. Zheng S. (2017). Inhibition of nonsense-mediated RNA decay by ER stress. RNA 23, 378–394. 10.1261/rna.058040.116 27940503 PMC5311500

[B43] Lim S. Y. M. Alshagga M. Kong C. Alshawsh M. A. Alshehade S. A. Pan Y. (2022). CYP35 family in *Caenorhabditis elegans* biological processes: fatty acid synthesis, xenobiotic metabolism, and stress responses. Arch. Toxicol. 96, 3163–3174. 10.1007/s00204-022-03382-3 36175686

[B44] Longman D. Plasterk R. H. A. Johnstone I. L. Caceres J. F. (2007). Mechanistic insights and identification of two novel factors in the *C. elegans* NMD pathway. Genes and Dev. 21, 1075–1085. 10.1101/gad.417707 17437990 PMC1855233

[B45] Ma L. Horvitz H. R. (2009). Mutations in the *Caenorhabditis elegans* U2AF large subunit UAF-1 alter the choice of a 3′ splice site *in vivo* . PLoS Genet. 5, e1000708. 10.1371/journal.pgen.1000708 19893607 PMC2762039

[B46] Martin L. Gardner L. B. (2015). Stress-induced inhibition of nonsense-mediated RNA decay regulates intracellular cystine transport and intracellular glutathione through regulation of the cystine/glutamate exchanger SLC7A11. Oncogene 34, 4211–4218. 10.1038/onc.2014.352 25399695 PMC4433865

[B47] Merret R. Nagarajan V. K. Carpentier M.-C. Park S. Favory J.-J. Descombin J. (2015). Heat-induced ribosome pausing triggers mRNA co-translational decay in *Arabidopsis thaliana* . Nucleic Acids Res. 43, 4121–4132. 10.1093/nar/gkv234 25845591 PMC4417158

[B48] Mitrovich Q. M. Anderson P. (2000). Unproductively spliced ribosomal protein mRNAs are natural targets of mRNA surveillance in *C. elegans* . Genes Dev. 14, 2173–2184. 10.1101/gad.819900 10970881 PMC316897

[B49] Pakos‐Zebrucka K. Koryga I. Mnich K. Ljujic M. Samali A. Gorman A. M. (2016). The integrated stress response. EMBO Rep. 17, 1374–1395. 10.15252/embr.201642195 27629041 PMC5048378

[B50] Pettit A. P. Jonsson W. O. Bargoud A. R. Mirek E. T. Peelor F. F. Wang Y. (2017). Dietary methionine restriction regulates liver protein synthesis and gene expression independently of eukaryotic initiation factor 2 phosphorylation in mice. J. Nutr. 147, 1031–1040. 10.3945/jn.116.246710 28446632 PMC5443467

[B51] Rajanala S. H. Ringquist R. Cryns V. L. (2019). Methionine restriction activates the integrated stress response in triple-negative breast cancer cells by a GCN2- and PERK-independent mechanism. Am. J. Cancer Res. 9, 1766–1775. 31497357 PMC6726988

[B52] Richardson C. E. Kinkel S. Kim D. H. (2011). Physiological IRE-1-XBP-1 and PEK-1 signaling in *Caenorhabditis elegans* larval development and immunity. PLoS Genet. 7, e1002391. 10.1371/journal.pgen.1002391 22125500 PMC3219621

[B53] Rollins J. A. Shaffer D. Snow S. S. Kapahi P. Rogers A. N. (2019). Dietary restriction induces posttranscriptional regulation of longevity genes. Life Sci. Alliance 2, e201800281. 10.26508/lsa.201800281 31253655 PMC6600014

[B54] Ron D. Walter P. (2007). Signal integration in the endoplasmic reticulum unfolded protein response. Nat. Rev. Mol. Cell Biol. 8, 519–529. 10.1038/nrm2199 17565364

[B55] Rual J.-F. Ceron J. Koreth J. Hao T. Nicot A.-S. Hirozane-Kishikawa T. (2004). Toward improving *Caenorhabditis elegans* phenome mapping with an ORFeome-based RNAi library. Genome Res. 14, 2162–2168. 10.1101/gr.2505604 15489339 PMC528933

[B56] Saitoh Y. Katane M. Kawata T. Maeda K. Sekine M. Furuchi T. (2012). Spatiotemporal localization of d -amino acid oxidase and d -aspartate oxidases during development in *Caenorhabditis elegans* . Mol. Cell. Biol. 32, 1967–1983. 10.1128/MCB.06513-11 22393259 PMC3347411

[B57] Shaffer D. Rollins J. (2020). Fluorescent polysome profiling in *Caenorhabditis elegans* . Bio Protoc. 10, e3742. 10.21769/BioProtoc.3742 PMC7842706 33659402

[B58] Sikalidis A. K. Lee J.-I. Stipanuk M. H. (2011). Gene expression and integrated stress response in HepG2/C3A cells cultured in amino acid deficient medium. Amino Acids 41, 159–171. 10.1007/s00726-010-0571-x 20361218 PMC3119335

[B59] Sikalidis A. K. Stipanuk M. H. (2010). Growing rats respond to a sulfur amino acid-deficient diet by phosphorylation of the alpha subunit of eukaryotic initiation factor 2 heterotrimeric complex and induction of adaptive components of the integrated stress response. J. Nutr. 140, 1080–1085. 10.3945/jn.109.120428 20357079 PMC2869497

[B60] Somers H. M. Fuqua J. H. Bonnet F. X. A. Rollins J. A. (2022). Quantification of tissue-specific protein translation in whole *C. elegans* using O-propargyl-puromycin labeling and fluorescence microscopy. Cell Rep. Methods 2, 100203. 10.1016/j.crmeth.2022.100203 35497499 PMC9046455

[B61] Sonenberg N. Hinnebusch A. G. (2009). Regulation of translation initiation in eukaryotes: mechanisms and biological targets. Cell 136, 731–745. 10.1016/j.cell.2009.01.042 19239892 PMC3610329

[B62] Teske B. F. Wek S. A. Bunpo P. Cundiff J. K. McClintick J. N. Anthony T. G. (2011). The eIF2 kinase PERK and the integrated stress response facilitate activation of ATF6 during endoplasmic reticulum stress. MBoC 22, 4390–4405. 10.1091/mbc.e11-06-0510 21917591 PMC3216664

[B63] Usuki F. Fujimura M. Yamashita A. (2013). Endoplasmic reticulum stress preconditioning attenuates methylmercury-induced cellular damage by inducing favorable stress responses. Sci. Rep. 3, 2346. 10.1038/srep02346 23907635 PMC3731649

[B64] Verfaillie T. Rubio N. Garg A. D. Bultynck G. Rizzuto R. Decuypere J.-P. (2012). PERK is required at the ER-mitochondrial contact sites to convey apoptosis after ROS-based ER stress. Cell Death Differ. 19, 1880–1891. 10.1038/cdd.2012.74 22705852 PMC3469056

[B65] Wanders D. Stone K. P. Forney L. A. Cortez C. C. Dille K. N. Simon J. (2016). Role of GCN2-independent signaling through a noncanonical PERK/NRF2 pathway in the physiological responses to dietary methionine restriction. Diabetes 65, 1499–1510. 10.2337/db15-1324 26936965 PMC4878423

[B66] Wang M. Kaufman R. J. (2016). Protein misfolding in the endoplasmic reticulum as a conduit to human disease. Nature 529, 326–335. 10.1038/nature17041 26791723

[B67] Wek R. C. Cavener D. R. (2007). Translational control and the unfolded protein response. Antioxidants Redox Signal. 9, 2357–2371. 10.1089/ars.2007.1764 17760508

[B68] Xiong H. Pears C. Woollard A. (2017). An enhanced *C. elegans* based platform for toxicity assessment. Sci. Rep. 7, 9839–9911. 10.1038/s41598-017-10454-3 28852193 PMC5575006

[B69] Zorio D. A. Blumenthal T. (1999). Both subunits of U2AF recognize the 3’ splice site in *Caenorhabditis elegans* . Nature 402, 835–838. 10.1038/45597 10617207

