# Supplementary figures and images for: The integrated stress response protects against ER stress but is not required for altered translation and lifespan from dietary restriction in Caenorhabditis elegans
Source: Front Cell Dev Biol. 2023 Dec 14;11:1263344. doi: 10.3389/fcell.2023.1263344 (PMC10755965; doi:10.3389/fcell.2023.1263344)

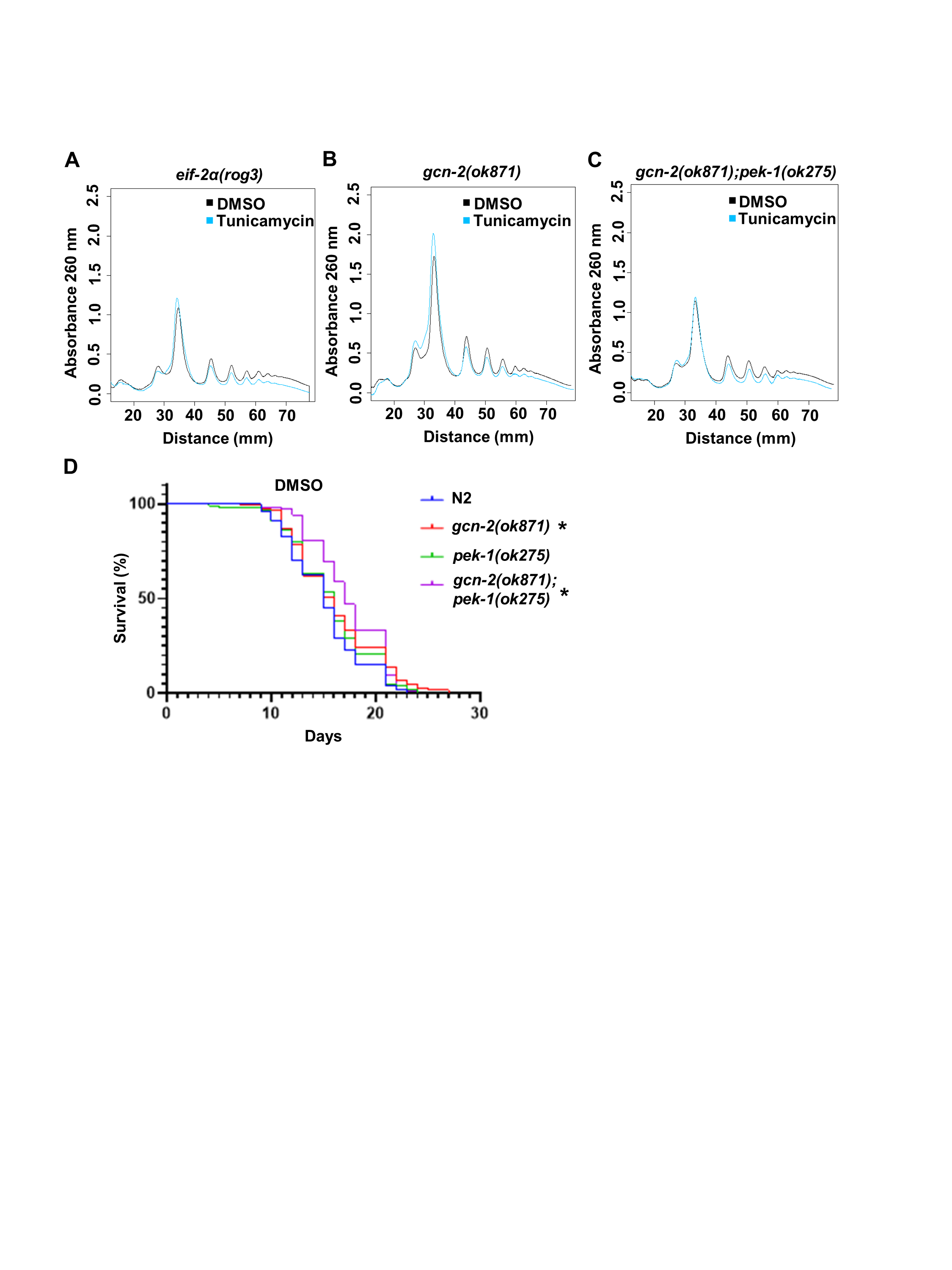

Supplement: Supplementary file 1 [file Image6.TIF]

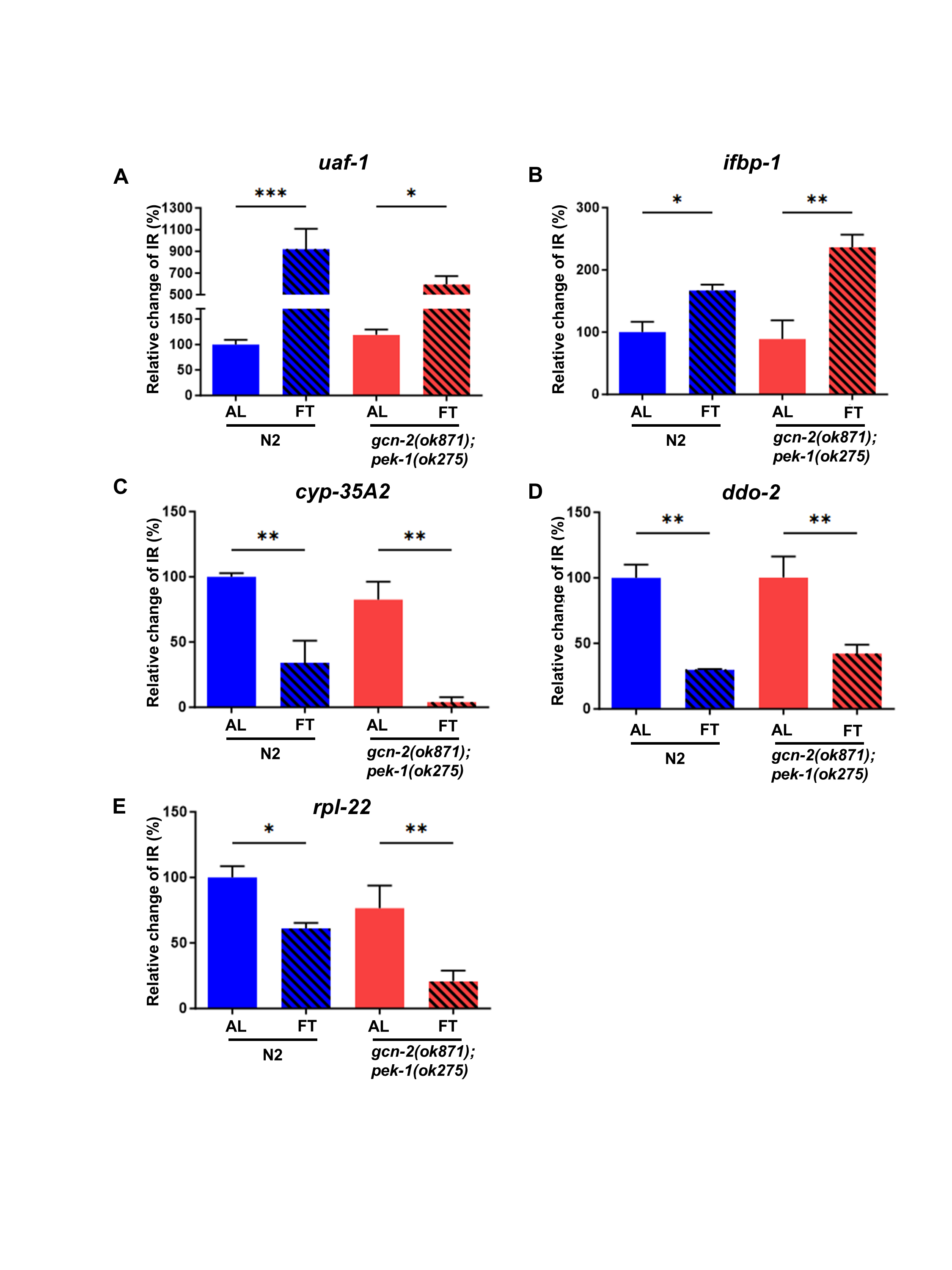

Supplement: Supplementary file 2 [file Image3.TIF]

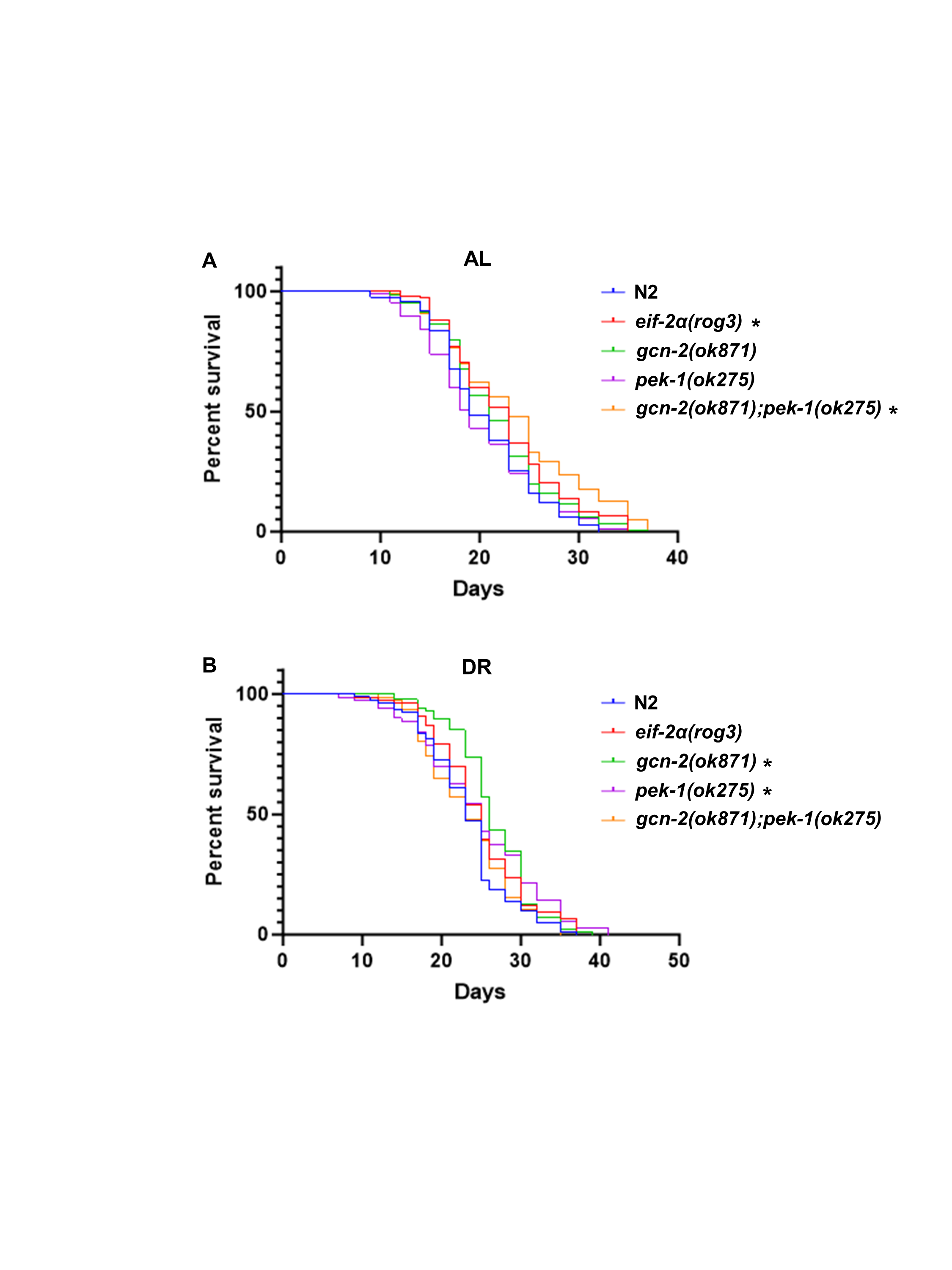

Supplement: Supplementary file 3 [file Image4.TIF]

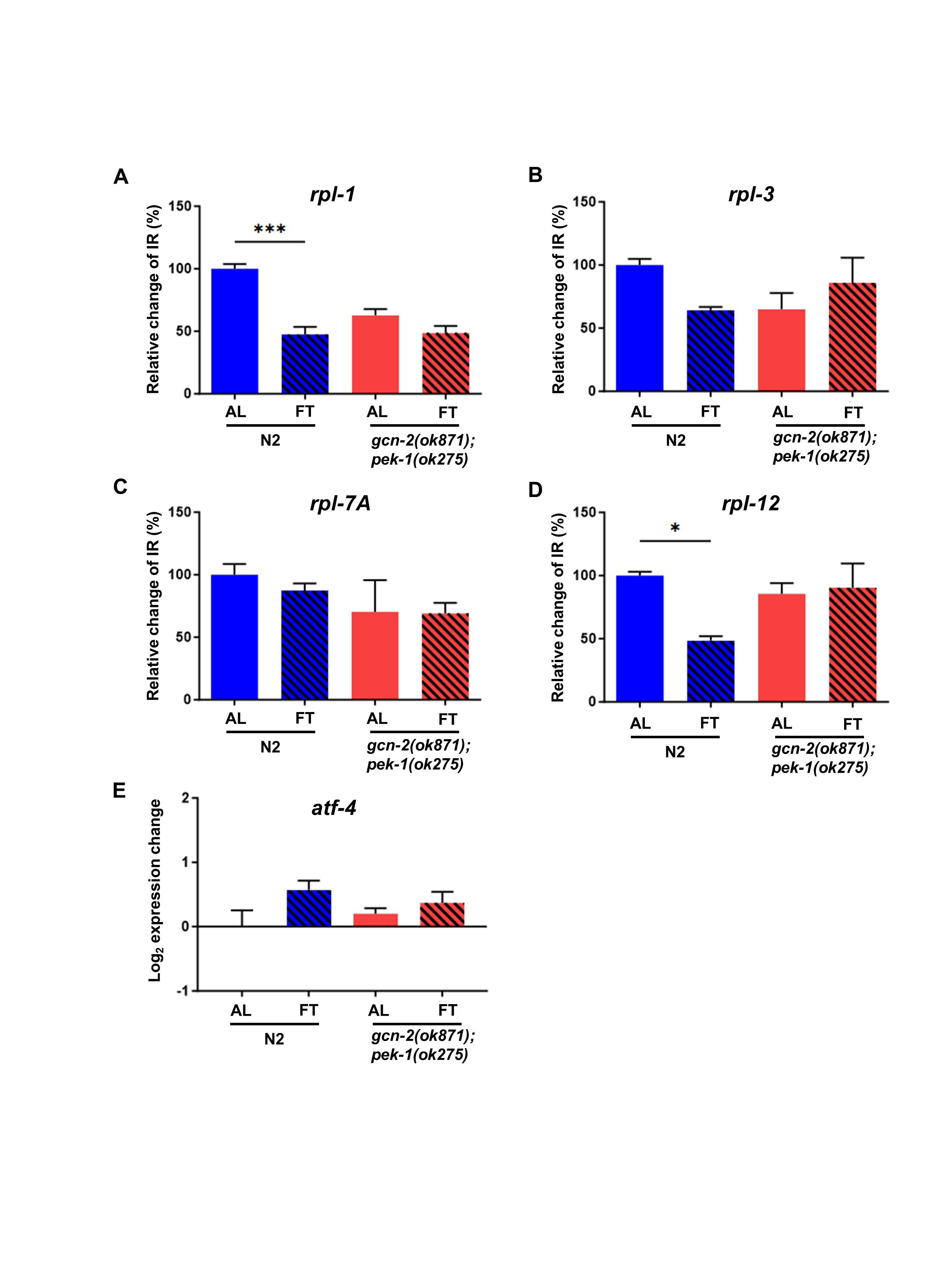

Supplement: Supplementary file 4 [file Image2.TIF]

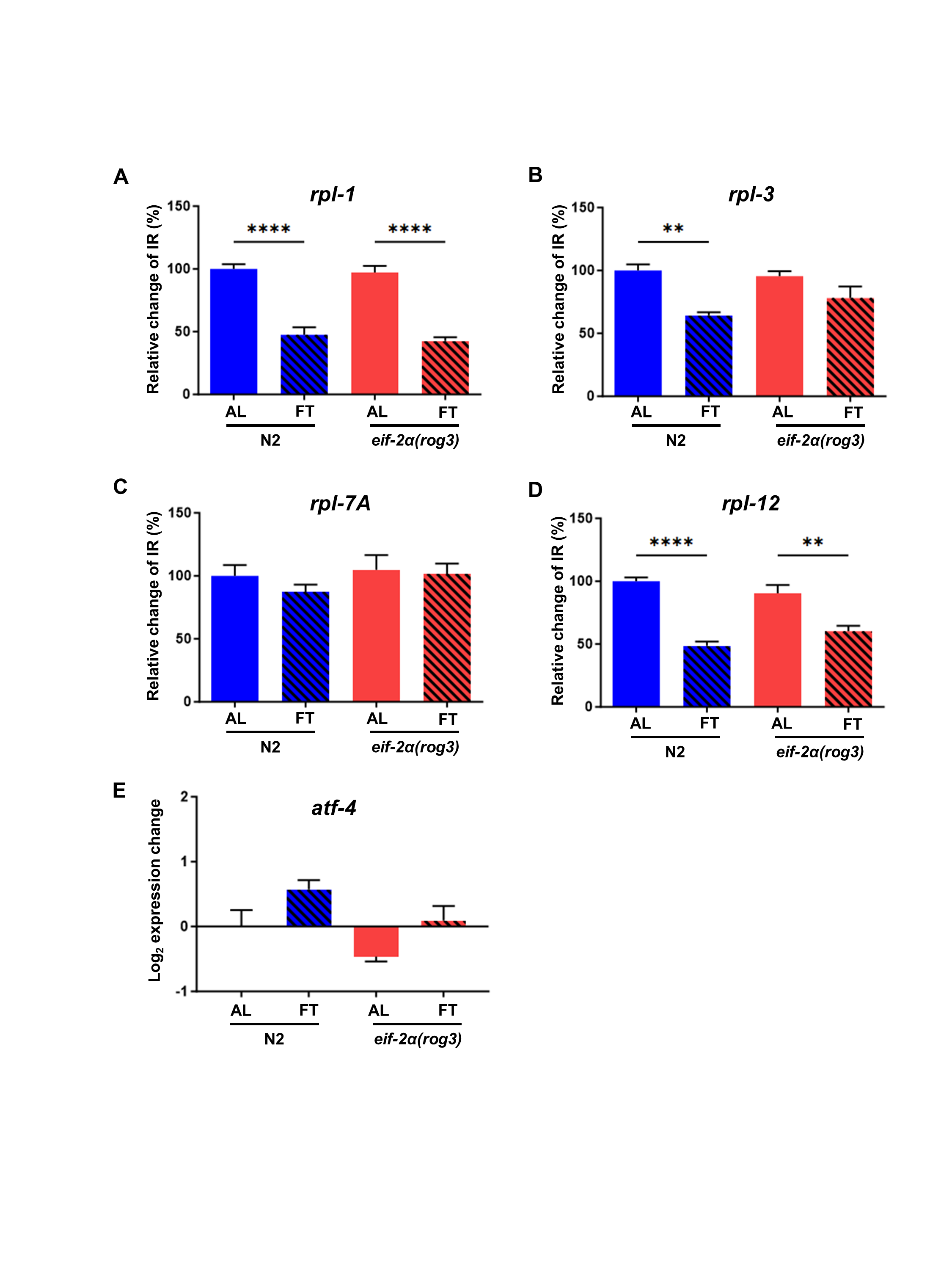

Supplement: Supplementary file 5 [file Image1.TIF]

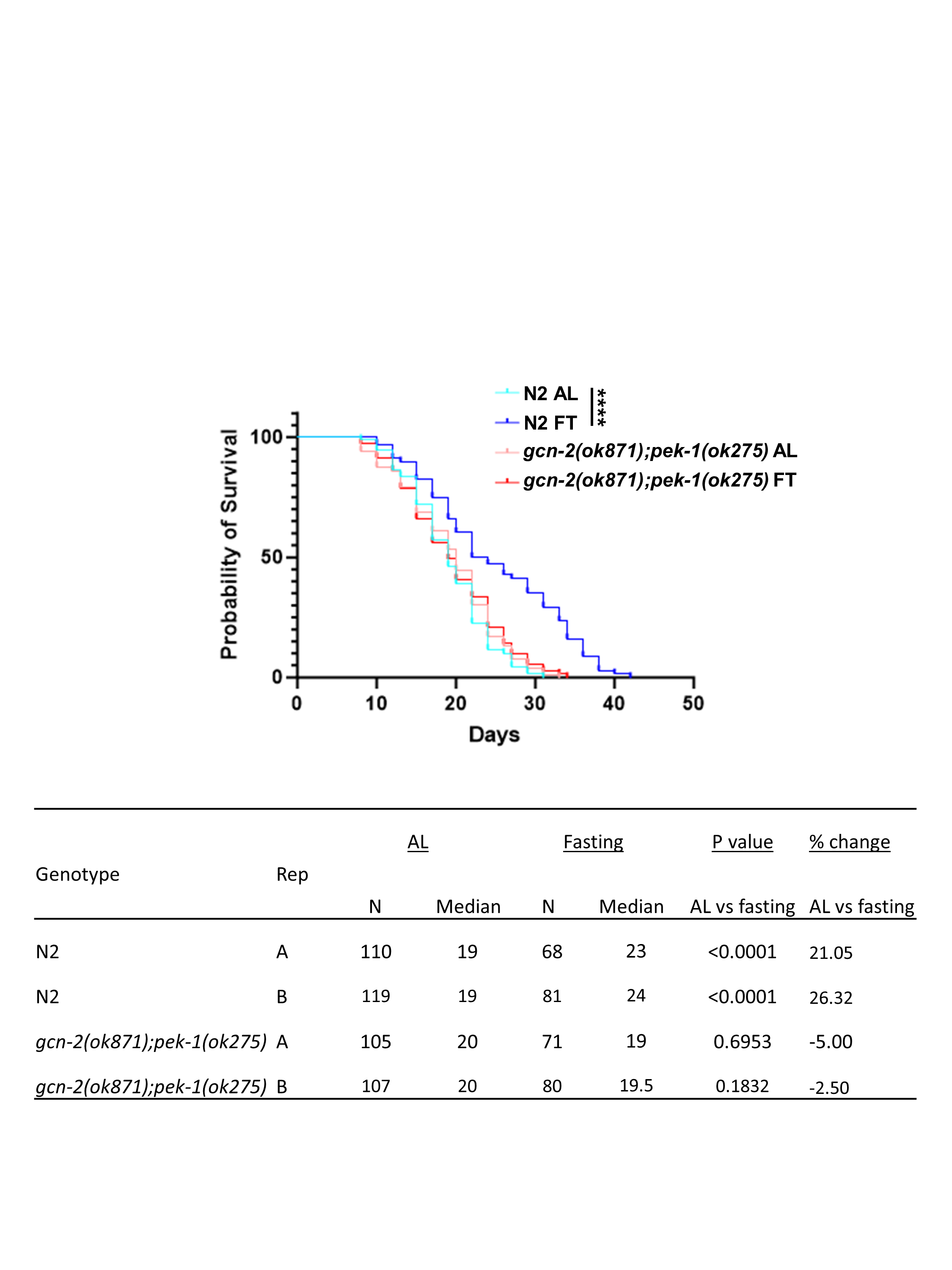

Supplement: Supplementary file 6 [file Image5.TIF]
