# Supplementary material for: The integrated stress response protects against ER stress but is not required for altered translation and lifespan from dietary restriction in Caenorhabditis elegans
Source: Front Cell Dev Biol. 2023 Dec 14;11:1263344. doi: 10.3389/fcell.2023.1263344 (PMC10755965; doi:10.3389/fcell.2023.1263344)
Supplement: Supplementary file 7 [file DataSheet1.docx]

**Supplemental Tables**

**Supplementary Table 1. List of strains used.**

| Strain name | Genotype | Backcrossed | Source |
| --- | --- | --- | --- |
| N2 | Wild type | n/a | CGC |
| DC19 | *bus-5(br19)* | n/a | CGC |
| ANR189 | *gcn-2(ok871)* | 3× | This study |
| ANR190 | *pek-1(ok275)* | 3× | This study |
| ANR191 | *gcn-2(ok871);pek-1(ok275)* | 3× | This study |
| ANR196 | *eif-2α(rog3)* | 3× | This study |
| JAR049 | *bus-5(br19);eif-2α(rog3)* | n/a | This study |
| PTCXi | *sec-23p::GFP::lacZ* with PTC | n/a | This study |
| ANR183 | *sec-23p::GFP::lacZ* with PTC; *eif-2α(rog3)* | n/a | This study |

**Supplementary Table 2. List of primers used.**

| Gene | Isoform(s) detected | Forward or reverse | Sequence |
| --- | --- | --- | --- |
| *uaf-1* | All | Forward | CACCTCTGTGGATTGGCTCA |
| *uaf-1* | All | Reverse | TTCCGTCAAATGCCATTCCG |
| *uaf-1* | Intron retention | Forward | TTGTCGTCGATTCGGCCAAT |
| *uaf-1* | Intron retention | Reverse | ATTTGGGCTTCCAGTTGGGA |
| *ifbp-1* | All | Forward | AGTCGGATCAGCAATGCCAT |
| *ifbp-1* | All | Reverse | AGCAAGACCGTGAGCTTCTC |
| *ifbp-1* | Intron retention | Forward | ATCACCCGCATTTTGCATGG |
| *ifbp-1* | Intron retention | Reverse | GTTGTTGTGGAGGCTGGAGA |
| *cyp-35A2* | All | Forward | GGAGTGCTCAAGAGACTGCA |
| *cyp-35A2* | All | Reverse | CGCGTCAGTGTAATCTTGCAG |
| *cyp-35A2* | Intron retention | Forward | AGCCGGTTTCGATTATAGAGGT |
| *cyp-35A2* | Intron retention | Reverse | TCTTGCCAGTGATCACCGTT |
| *ddo-2* | All | Forward | CCACCAAGCAGGACAATCGA |
| *ddo-2* | All | Reverse | TGTCCTCTTCTGCGCTTCAA |
| *ddo-2* | Intron retention | Forward | TCCAAAGTGAGTAGAAGCCGA |
| *ddo-2* | Intron retention | Reverse | TCTGACATGCTTACGTCCCG |
| *rpl-22* | All | Forward | AAGGTCTCAGTCGTCTCCGA |
| *rpl-22* | All | Reverse | GCCAATCACGGAGGGAGTTT |
| *rpl-22* | Intron retention | Forward | TGCACCTCAAGTTCAACGTTG |
| *rpl-22* | Intron retention | Reverse | TACCCTAAGCCTGGTCTTGC |
| *rpl-1* | All | Forward | GCCACCGTCAAGTTCCAGAT |
| *rpl-1* | All | Reverse | CTCCTCCTGGGTGAGTCCAA |
| *rpl-1* | Intron retention | Forward | ACAGAAGGACAAGCGTTTCA |
| *rpl-1* | Intron retention | Reverse | GTGCTTCAGTATTATACCAGCTC |
| *rpl-3* | All | Forward | CCAGAAGACCATCACCCCAA |
| *rpl-3* | All | Reverse | AGACGCTTCTTTGGTCCGAG |
| *rpl-3* | Intron retention | Forward | ACAGCAACTGTCGCGTTTC |
| *rpl-3* | Intron retention | Reverse | GGACGCTTTAGCCACTCAGT |
| *rpl-7a* | All | Forward | GAAAGGCTTCCCTCGGAACC |
| *rpl-7a* | All | Reverse | GGTCTCGACGAGCTTGTTCA |
| *rpl-7a* | Intron retention | Forward | AGCCAGTCCGGTTGGTAT |
| *rpl-7a* | Intron retention | Reverse | AGGCTTGTCTAGTTCACCTATC |
| *rpl-12* | All | Forward | GAGACCAAGATCGATGGCCA |
| *rpl-12* | All | Reverse | TGATGTCGTGTGGGTGTTGT |
| *rpl-12* | Intron retention | Forward | GTGATTTTCATGCTCCTGAAG |
| *rpl-12* | Intron retention | Reverse | CAAGGATCTCCTTGACGG |
| *atf-4* | N/A | Forward | TCCAAATGGTTCCACAAAGC |
| *atf-4* | N/A | Reverse | GCGCACGATTTCTTCGAG |

**Supplementary Table 3. Survival of the indicated strains under AL or DR.**

| Genotype | Rep | AL  Animals Median | | DR  Animals Median | | *P* value AL vs DR | % change AL vs DR |
| --- | --- | --- | --- | --- | --- | --- | --- |
| N2 | A | 114 | 19 | 80 | 23 | 0.0006 | 21.05 |
| N2 | B | 93 | 21 | 60 | 26 | <0.0001 | 23.81 |
| *eif-2α(rog3)* | A | 108 | 23 | 76 | 25 | 0.0095 | 8.70 |
| *eif-2α(rog3)* | B | 98 | 24 | 49 | 26 | 0.0632 | 8.33 |
| *gcn-2(ok871)* | A | 127 | 21 | 87 | 26 | <0.0001 | 23.81 |
| *gcn-2(ok871)* | B | 109 | 21 | 64 | 24 | 0.0005 | 14.29 |
| *pek-1(ok275)* | A | 107 | 19 | 70 | 25 | <0.0001 | 31.58 |
| *pek-1(ok275)* | B | 94 | 22 | 70 | 28 | <0.0001 | 27.27 |
| *gcn-2(ok871); pek-1(ok275)* | A | 119 | 23 | 77 | 23 | 0.3621 | 0.00 |
| *gcn-2(ok871); pek-1(ok275)* | B | 85 | 22 | 75 | 21 | 0.1239 | -4.55 |

**Supplementary Table 4. Survival statistics of the heat stress recovery assay.**

| Rep | animals in N2 | survival after 5 days in N2 | % survival in N2 | animals in *eif-2α(rog3)* | survival after 5 days in  *eif-2α(rog3)* | % survival in *eif-2α(rog3)* | *P* value |
| --- | --- | --- | --- | --- | --- | --- | --- |
| A | 84 | 10 | 11.90 | 87 | 28 | 32.18 | 0.0098 |
| B | 99 | 16 | 16.16 | 98 | 37 | 37.76 | <0.0001 |
| C | 104 | 16 | 15.38 | 101 | 37 | 36.63 | <0.0001 |

**Supplementary Table 5. Survival statistics under fasting conditions.**

| Genotype | Rep | AL  N Median | | Fasting  N Median | | *P* value  AL vs fasting | % change  AL vs fasting |
| --- | --- | --- | --- | --- | --- | --- | --- |
| N2 | A | 84 | 19 | 86 | 22 | <0.0001 | 15.79 |
| N2 | B | 95 | 20 | 88 | 25 | <0.0001 | 25.00 |
| *eif-2α(rog3)* | A | 95 | 26 | 66 | 30 | <0.0001 | 15.38 |
| *eif-2α(rog3)* | B | 100 | 26 | 68 | 29 | <0.0001 | 11.54 |

**Supplementary Table 6. Survival statistics of eIF-2α phospho-mutant under ER stress conditions.**

| Genotype | Rep | DMSO  N Median | | Tunicamycin  N Median | | *P* value  DMSO vs Tuni | % change  DMSO vs Tuni |
| --- | --- | --- | --- | --- | --- | --- | --- |
| N2 | A | 89 | 19 | 91 | 15 | <0.0001 | -21.05 |
| N2 | B | 96 | 19 | 94 | 15 | <0.0001 | -21.05 |
| *eif-2α(rog3)* | A | 92 | 19 | 88 | 13 | <0.0001 | -31.58 |
| *eif-2α(rog3)* | B | 88 | 20 | 99 | 12 | <0.0001 | -40.00 |

**Supplementary Table 7. Survival statistics of ISR kinase mutants under ER stress conditions.**

| Genotype | Rep | DMSO  N Median | | Tunicamycin  N Median | | *P* value  DMSO vs Tuni | % change  DMSO vs Tuni |
| --- | --- | --- | --- | --- | --- | --- | --- |
| N2 | A | 104 | 15 | 103 | 7 | <0.0001 | -53.33 |
| N2 | B | 113 | 14 | 82 | 4 | <0.0001 | -71.42 |
| *gcn-2(ok871)* | A | 124 | 16 | 116 | 9 | <0.0001 | -43.75 |
| *gcn-2(ok871)* | B | 90 | 14 | 85 | 8 | <0.0001 | -42.86 |
| *pek-1(ok275)* | A | 108 | 16 | 122 | 3 | <0.0001 | -81.25 |
| *pek-1(ok275)* | B | 102 | 13 | 83 | 2 | <0.0001 | -84.62 |
| *gcn-2(ok871); pek-1(ok275)* | A | 102 | 17 | 118 | 2 | <0.0001 | -88.24 |
| *gcn-2(ok871); pek-1(ok275)* | B | 92 | 13 | 107 | 2 | <0.0001 | -84.62 |

**Supplemental figure legends**

**Supplementary Figure 1.** Intron retained (IR) isoform expression of *rpl-1* (A), *rpl-3* (B), *rpl-7A* (C), *rpl-12* (D), and the expression of *atf-4* (E) in N2 and *eif-2α(rog3)* after 3 days of AL or fasting (FT) in young adult worms. IR expression was normalized by expression of all isoforms of each gene. Error bars represent means ± SEM (n=3). One-way ANOVA with Dunnett’s post hoc test was performed. * *P <0.05;* ** *P <0.01;* *** *P <0.001; and* **** *P <0.0001*.

**Supplemental Figure 2.** Intron retained (IR) isoform expression of *rpl-1* (A), *rpl-3* (B), *rpl-7A* (C), *rpl-12* (D), and the expression of *atf-4* (E) in N2 and *gcn-2(ok871);pek-1(ok275)* after 3 days of AL or fasting (FT) in young adult worms. IR expression was normalized by expression of all isoforms of each gene. Error bars represent means ± SEM (n=3). One-way ANOVA with Dunnett’s post hoc test was performed. * *P <0.05;* ** *P <0.01;* *** *P <0.001; and* **** *P <0.0001*.

**Supplemental Figure 3.** Intron retained (IR) isoform expression of *uaf-1* (A), *ifbp-1* (B), *cyp-35A2* (C), *ddo-2* (D), and *rpl-22* (E) in N2 and *gcn-2(ok871);pek-1(ok275)* after 3 days of AL or fasting (FT) in young adult worms. IR expression was normalized by expression of all isoforms of each gene. Error bars represent means ± SEM (n=3). One-way ANOVA with Dunnett’s post hoc test was performed. * *P <0.05;* ** *P <0.01;* *** *P <0.001; and* **** *P <0.0001*.

**Supplemental Figure 4.** The lifespan of ISR mutants under AL (A) and DR (B). Kaplan–Meier survival curves were compared using the Mantel–Cox log-rank test. * *P <0.05* for each mutant compared to N2. See Fig. 4 for AL and DR comparisons for each strain. See Table S3 for additional details and replicates.

**Supplemental Figure 5.** Survival of worms under AL or fasting (FT) treatments starting from day 1 adulthood. Kaplan–Meier survival curves were compared using the Mantel–Cox log-rank test. **** *P <0.0001*. Two independent trials were conducted. The representative figure is shown.

**Supplementary Figure 6.** The effect of ISR in ER stress response. A-C) Polysome profiling of day 1 adult *eif-2α(rog3)* (A), *gcn-2(ok871)* (B) and *gcn-2(ok871);pek-1(ok871)* (C). Day 1 adult worms were treated with DMSO or 25 µg/mL tunicamycin for 3 h prior to cell lysis. D) Survival of worms in DMSO starting from day 1 adulthood. Kaplan–Meier survival curves were compared using the Mantel–Cox log-rank test. * *P <0.05;* ** *P <0.01;* *** *P <0.001; and* **** *P <0.0001*.
